# Time-resolved concentrations of serum amino acids, one-carbon metabolites and B-vitamin biomarkers during the postprandial and fasting state: the Postprandial Metabolism in Healthy Young Adults (PoMet) Study

**DOI:** 10.1017/S0007114523002490

**Published:** 2024-03-14

**Authors:** Åslaug Matre Anfinsen, Christina Osland Johannesen, Vilde Haugen Myklebust, Hanne Rosendahl-Riise, Adrian McCann, Ottar Kjell Nygård, Jutta Dierkes, Vegard Lysne

**Affiliations:** 1 Mohn Nutrition Research Laboratory, Centre for Nutrition, Department of Clinical Science, University of Bergen, Bergen, Norway; 2 Mohn Nutrition Research Laboratory, Centre for Nutrition, Department of Clinical Medicine, University of Bergen, Bergen, Norway; 3 Bevital AS, Bergen, Norway; 4 Department of Heart Disease, Haukeland University Hospital, Bergen, Norway; 5 Laboratory Medicine and Pathology, Haukeland University Hospital, Bergen, Norway

**Keywords:** Postprandial Response, Fasting, Metabolism, Metabolites, Metabolomics, Biomarkers, Epidemiology

## Abstract

Metabolomics has been utilised in epidemiological studies to investigate biomarkers of nutritional status and metabolism in relation to non-communicable diseases. However, little is known about the effect of prandial status on several biomarker concentrations. Therefore, the aim of this intervention study was to investigate the effect of a standardised breakfast meal followed by food abstinence for 24 h on serum concentrations of amino acids, one-carbon metabolites and B-vitamin biomarkers. Thirty-four healthy subjects (eighteen males and sixteen females) aged 20–30 years were served a breakfast meal (∼500 kcal) after which they consumed only water for 24 h. Blood samples were drawn before and at thirteen standardised timepoints after the meal. Circulating concentrations of most amino acids and metabolites linked to one-carbon metabolism peaked within the first 3 h after the meal. The branched-chain amino acids steadily increased from 6 or 8 hours after the meal, while proline decreased in the same period. Homocysteine and cysteine concentrations immediately decreased after the meal but steadily increased from 3 and 4 hours until 24 h. FMN and riboflavin fluctuated immediately after the meal but increased from 6 h, while folate increased immediately after the meal and remained elevated during the 24 h. Our findings indicate that accurate reporting of time since last meal is crucial when investigating concentrations of certain amino acids and one-carbon metabolites. Our results suggest a need for caution when interpretating studies, which utilise such biomarkers, but do not strictly control for time since the last meal.

The metabolome is dynamic and constantly changing in response to external stimuli. Dietary intake is one such external factor triggering notable metabolic and hormonal changes in the hours following food intake. For instance, postprandial concentrations of glucose and insulin change in response to the intake of specific food or nutrients^(1,2)^. Consequently, prandial status at the time of blood sampling is accounted for when defining the normal ranges of glucose and insulin^(3)^. The metabolome undergoes dynamic changes not only immediately after dietary intake but also during prolonged fasting. In 2011, Rubio-Aliaga and colleagues^(4)^ reported an analysis of the ‘human fasting metabolome’. In their study, ten healthy volunteers fasted for 36 h, and blood samples were taken at 12 and 36 h after the last meal. They observed that over 70 % of circulating amino acid concentrations changed during prolonged fasting, with notable decreases in methionine and tryptophan and increases in the branched-chain amino acids (BCAA). In 2012, Krug *et al.* ^(5)^ investigated metabolite changes in response to several metabolic challenges, including 36 h of fasting in fifteen healthy males. Like Rubio-Aliaga *et al*., they reported that the BCAA and NEFA concentrations increased during 36 h of fasting. Teruya *et al.* ^(6)^ investigated a range of metabolites during 58 h of fasting in a small study of four participants. Similarly, they also reported increased concentrations of BCAA in addition to changes in butyrates, acylcarnitines, some co-enzymes and other amino acids.

Circulating metabolite concentrations are frequently investigated in epidemiological studies as predictors or mediators of health and disease outcomes. For instance, homocysteine concentrations have been extensively investigated as a risk factor for a variety of diseases including CVD^(7)^ and dementia^(8)^. High concentrations of the BCAA are also reported as biomarkers for increased risk of diabetes^(9)^. However, there are several potential sources of measurement error that should be considered when evaluating blood biomarker concentrations in epidemiological studies. Such measurement errors are not limited to sample processing and laboratory analyses but may also arise from the within-person variability of the biomarker. As the concentrations of blood metabolites may fluctuate within subjects in response to dietary intake and prandial status, a single measurement of the biomarker may be a poor measure of the true aetiologic exposure^(10)^. To limit the impact of prandial status, it is common in epidemiological studies to apply a distinct cut-off to distinguish between the postprandial and the fasting state. This may be convenient, but the transition between the two states exists on a continuum with no clear cut-off for being in one state or the other. Further, the duration of the postprandial state is influenced by factors such as meal size and composition. Smaller meals high in simple carbohydrates are typically digested and absorbed more rapidly, resulting in a postprandial period of approximately 2–3 h. On the other hand, larger meals that are rich in fats can extend the postprandial period up to 8 h^(11,12)^. In the literature, some use 90 min^(13)^, 2 h^(14)^, 3 h^(15)^, 4 h^(16)^ or 6 h^(17)^ to define the postprandial state. It is evident that the duration of the postprandial period is challenging to define precisely due to its variability, and that applying a cut-off at a certain time after a meal may not sufficiently account for the fluctuating nature of the metabolome in response to dietary intake and fasting.

Although previous studies have demonstrated metabolic changes linked to the postprandial and fasting states, most studies tend to commence blood sampling after overnight fast^(4–6)^ or only during the first few hours after a meal^(18)^. Data on the metabolic changes during the adaption from the postprandial to the fasting state are missing. Therefore, the aim of this study was to investigate how serum concentrations of amino acids, one-carbon metabolites and B-vitamin biomarkers change during the 24 h after a standardised breakfast meal in healthy, young individuals.

## Methods

### Recruitment and pre-screening

Information about the study was spread through social media channels and posters in the nearby area of Bergen, Norway during the summer of 2021. Individuals who were interested in participating in the study were contacted and pre-screened over the phone, and individuals eligible for inclusion were invited to the main screening and to attend the study visit. Overall, the aim was to recruit young, healthy participants. The inclusion criteria were: (1) aged 20–30 years (born 1991–2001); (2) self-reported BMI 22–27 kg/m^2^ at phone screening, while subjects were excluded if they (1) had experienced acute or chronic disease such as diabetes, thyroid diseases, cancer, CVD or inflammatory bowel disease during the last 3 years; (2) had celiac disease or other food allergies interfering with the standardised breakfast meal; (3) used any prescription medications except for contraceptives; (4) smoked or used other nicotine-containing products such as ‘snuff’ regularly; (5) had been pregnant or breastfed the last 3 months before study visit and (6) had experienced weight change > 5 % during the last 3 months before the study visit. The inclusion and exclusion criteria for participation in the study are summarised in Table 1.

**Table 1.** Inclusion and exclusion criteria for participation in the Postprandial Metabolism Study

Inclusion criteria	Exclusion criteria
• Aged 20–30 years (birth years 1991–2001) • Self-reported BMI 22–27 kg/m^2^ at phone screening	• Acute or chronic disease such as diabetes, thyroid diseases, cancer, CV or inflammatory bowel disease during the last 3 years • Celiac disease or other food allergies interfering with the standardised breakfast meal • Use of any prescription medications except contraceptives • Smoking or regular use of other nicotine-containing products, such as ‘snuff’ • Pregnancy of breast-feeding the last 3 months before the study visit • Significant weight change (> 5 %) during the last 3 months before the study visit

### Instructions before the study visit

To standardise physiological and metabolic conditions, all individuals were instructed to (1) not use dietary supplements the last 7 d before the visit; (2) abstain from smoking and use of nicotine-containing products such as ‘snuff’ the last 7 d before the visit; (3) abstain from alcohol and avoid any strenuous activity the last 24 h before the visit; (4) consume an evening meal consisting of three slices of bread with cheese and jam, and a glass of juice at 20.00 the evening before the study visit; (5) not consume anything other than water after the evening meal before the study visit and (6) drink enough water and to stay hydrated to facilitate blood sampling and the insertion of a venous catheter.

### Study visits

**Figure 1.**
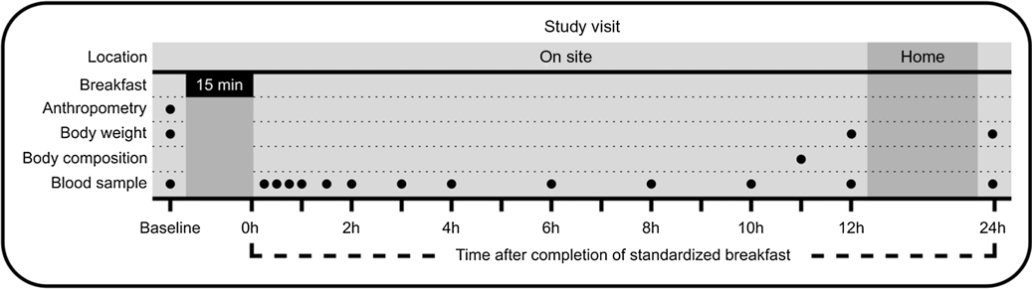
Sampling times in the Postprandial Metabolism Study.

The course of the study visits in this intervention study is illustrated in Fig. 1. The study was conducted at the Research Unit for Health Surveys, the University of Bergen, Norway. On the morning (between 07.30 and 08.00 hours) at the attendance of the study visit, all individuals had their height and body weight measured to calculate their BMI for screening purposes. The measurements were conducted by the same researcher at all study visits to ensure similar measurements. Height was measured to the nearest 0·1 cm using a Seca 217 stadiometer, with individuals standing without shoes and in light clothing, feet gathered and the head positioned in the Frankfurt horizontal plane. Body weight was measured and rounded to the nearest 0·1 kg using a Seca 877 flat scale, measured without shoes and in light clothing. Due to variation between scales (home *v*. study centre), clothing and hydration status when measuring body weight, some deviations from the BMI criteria (self-reported BMI between 22 and 27 kg/m^2^) at study entry were accepted. Participants who fulfilled all inclusion and exclusion criteria according to Table 1 were included in the study. Adherence to the instructions before the study visit was controlled by self-reported questionnaires, but participants were not excluded if they deviated from the instructions.

### Blood sampling and preanalytical handling of blood samples

A venous catheter was placed in the elbow cavity. Ten minutes after the insertion of the catheter, a venous blood sample and capillary blood glucose (Hemocue® Glucose 201 RT Analyzer) were taken. Thereafter, the standardised breakfast meal was served. After consumption of the meal, a total of thirteen blood samples were drawn as shown in Fig. 1. Blood samples were drawn particularly frequently during the first 4 h (timepoints 15 min, 30 min, 45 min, 60 min, 90 min, 2 h, 3 h and 4 h after the meal), and then every other hour until 12 h after the meal. After the 12-h blood sample, the participants left the study centre overnight and came back the next morning for the last blood sample taken 24 h after the breakfast meal (Fig. 1). Capillary blood glucose was also measured at each timepoint before the venous blood sampling. After the 10-h blood sample, the venous catheter was removed to facilitate the body composition analysis. Therefore, the 12- and 24-h blood samples were taken as normal venous blood samples. At each timepoint, a total of 11·5 ml of blood was drawn and distributed into serum tubes (8·5 ml, BD Vacutainer^®^ SST^TM^ II *Advance*; Becton, Dickinson, and Company) and EDTA tubes (3 ml, Vacuette^®^ K2EDTA). At baseline and at the 24-h timepoint, an additional 6 ml and 3 ml EDTA blood was collected, respectively, for measurement of haematology and safety biomarkers (aminotransferases, creatinine, C-reactive protein, erythrocytes, gamma-glutamyltransferase, Hb, hBA1c, mean corpuscular Hb, mean corpuscular volume, mean platelet volume, thrombocytes, thyroid stimulating hormone and 25-hydroxyvitamin D).

After the blood sampling, the serum tubes were stored at room temperature for 30–60 min and then centrifuged at 2200 × ***g*** for 10 min at 20°C. EDTA tubes were centrifuged within 15 min after collection, at 2200× ***g*** for 10 min at 4°C. Three aliquots of serum and one aliquot of plasma per timepoint were temporarily stored in a freezer at −20°C and transferred to −80°C at the end of the day. Additionally, one aliquot of serum from each time point was stored in the refrigerator at +4°C and transported to the laboratory daily, together with the additional EDTA blood collected at baseline and the 24 h timepoint.

### Breakfast meal

The breakfast meal consisted of wholegrain wheat bread with butter, strawberry jam, low-fat cheese (16 % fat), cucumber and a glass of orange juice. The amounts and nutrient composition of the breakfast meal are given in Table 2. The breakfast meal was composed to mimic a normal Norwegian breakfast and provided 20–24 % of the daily energy needs, which are estimated to be 2600 kcals and 2150 kcal per day for inactive males and females, respectively^(19)^. All participants were instructed to consume the breakfast in precisely 15 min, and the minute the last bite was consumed was set to timepoint zero. After the breakfast meal was consumed, the participants were instructed not to consume anything other than water (no chewing gum, sparkling water, diet soda, etc.) for the next 24 h.

**Table 2.** The breakfast meal in the Postprandial Metabolism Study*

Food item	Wheat bred	Butter	Low-fat cheese	Strawberry jam	Orange juice	Cucumber	Total
Grams	90	15	40	20	200	36	401 g
Energy (kcal)	213	81	107	26	82	4	513 kcal
Macronutrients							
Fat (g)	4·7	9	6·4	0	0·4	0	20·5 g (35·9 E%)
Carbohydrate (g)	34·5	0·1	0	6·2	18·2	0·4	59·4 g (46·3 E%)
Dietary fibre (g)	4·0	0	0	0·3	0·2	0·3	4·8 g (1·9 E%)
Protein (g)	6·2	0·1	12·4	0·1	1·4	0·3	20·5 g (16·0 E%)
B-vitamins†							
Thiamine (mg)	0·27	0	0·1	0	0·16	0·01	0·54 mg
Riboflavin (mg)	0·07	0	0·1	0	0·04	0·01	0·22 mg
Niacin (mg)	2	0	0·8	0	0·4	0·1	3·3 mg
Vitamin B_6_ (mg)	0·09	0	0·02	0·01	0·1	0·01	0·23 mg
Folate (µg)	29	0	17	3	56	4	109 µg
Vitamin B_12_ (µg)	0	0	1	0	0	0	1 µg

* The same breakfast was provided to all participants irrespective of body weight or sex.

† The B-vitamins were estimated using ‘Matvaretabellen’ (www.matvaretabellen.no), a tool developed by the Norwegian Food Safety Authority and the Norwegian Directorate of Health.

### Body composition analysis

After the 10-h blood sample, body composition was analysed using a BodPod (COSMED, version 5.4.6). To standardise measurements, the analysis was conducted by the same researcher at all study visits, and all participants were instructed not to consume any water for the last 2 h before the measurement. The analysis was carried out with participants wearing swimwear or underwear of synthetic material and a swimming cap, and wearing no jewellery or piercings.

### Laboratory analyses

The measurement of capillary glucose was performed using a handheld device (Hemocue® Glucose 201 RT Analyzer). All amino acids and one-carbon metabolites were analysed in serum at Bevital AS. Alanine, asparagine, aspartic acid, cystathionine, cysteine, glutamic acid, glutamine, glycine, histidine, total homocysteine, isoleucine, leucine, lysine, methionine, phenylalanine, proline, sarcosine, serine, threonine, tryptophan, tyrosine and valine were analysed using a gas-chromatography mass spectrometry (GS-MS/MS), while arginine, betaine, choline, dimethylglycine and methionine sulfoxide were analysed using a liquid-chromatography mass spectrometry (LC-MS/MS). Among the B-vitamin biomarkers, serum cobalamin and total serum folate (i.e. the sum of 5-methyltetrahydrofolate, 5-formyltetrahydrofolate and pteroylglutamic acid) were analysed at the Department of Medical Biochemistry and Pharmacology at Haukeland University Hospital, Bergen, Norway (certified NS-EN ISO 15189:2012) using immunoassay. FMN, N1´-methylnicotinamide, nicotinamide, pyridoxal, pyridoxal-5-phosphate, 4´-pyridoxic acid, riboflavin, thiamine and thiamine monophosphate (TMP) were analysed at Bevital AS using an LC-MS/MS, while methylmalonic acid was analysed using a GC-MS/MS. An overview of the metabolites and safety markers measured, and their analytical methods are found in online Supplementary Table 1.

### Quality assurance

Standardised operating procedures were developed and followed throughout the study to ensure accurate and similar measurements. Qualified personnel conducted the blood sampling, and efforts were made to have the same staff member carry out data collection on the same participant to avoid systematic differences in data collection.

### Statistical analyses

All statistical analyses were performed using R version 4.1.3 (R Foundation for Statistical Computing, https://www.r-project.org/) and the packages within the *tidyverse* and *irrICC*.

Negative values are not possible with biological data. Further, most biomarkers are skewed with a longer tail towards higher values. Therefore, all metabolite concentrations were log-transformed before statistical analysis and described using the back-transformed gMean and gSD as recommended^(20,21)^. Descriptive statistics are supplemented with ranges (min–max). Inferential statistics are accompanied by 95 % geometric compatibility (confidence) intervals (gCI) as a measure of uncertainty, calculated using the geometric standard error (gSE) and formulas 1–3:
(1)

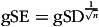



(2)

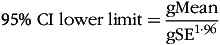



(3)

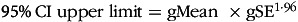




The main objective is presented visually, by plotting the raw metabolite concentrations as a function of time, with the mean time-course indicated by superimposing the geometric mean concentrations (95 % gCI) on top of the individual data. Relative changes in metabolite concentrations were calculated for each individual, with each pre-breakfast blood sample utilised as an individual reference value. These individual percentage changes were subsequently combined to calculate the gMean percentage change across the study cohort. To evaluate the degree to which the different biomarkers are affected during the postprandial period, the within-person reproducibility was quantified by calculating intra-class correlation coefficients (ICC) on log-transformed data. The ICC were calculated on the basis of a two-way random-effects model for absolute agreement, using the *irrlCC* package and the function *icc2.nointer.fn()* ^(22)^.

### Sample size calculation

The sample size calculation was performed using an accuracy-in-parameter-estimation approach, as recommended when the main purpose is to accurately estimate the parameters of interest^(20,21)^. For the main analysis, we aimed to achieve a multiplicative margin-of-error (gSE1·96) < 1·10, corresponding to a gSE < 1·05, for at least 80 % of the measurements. Using freely available data on 132 metabolites across fifty-six timepoints (7392 estimates) across different metabolic challenges from the HuMet study^(5)^ (available from http://metabolomics.helmholtz-muenchen.de/humet/), the observed median (80th percentile) gSD was 1·24 (1·32). Rearranging equation 1 above, and solving for *n* with a gSD = 1·32, we needed a sample size of 32 to achieve the desired precision level. Precision curves as a function of sample size are provided in online Supplementary Fig. 1(a). The expected distribution of multiplicative margin-of-errors with a sample size of 32 is illustrated in online Supplementary Fig. 1(b), based on repeated resampling with replacement from HuMet (50 replications, 369 600 simulated estimates). We expected a dropout of up to 10 % due to adverse events following fasting blood sampling or difficulties drawing blood from a venous catheter. Therefore, to achieve our goal of collecting complete data for thirty-two participants, we aimed to recruit a total of thirty-six participants (eighteen males and eighteen females).

### Ethics and safety

The study was registered at ClinicalTrials.gov (NCT number 04989478). The study was conducted according to the guidelines laid down in the Declaration of Helsinki, and all procedures involving human subjects were approved by The Regional Committee for Health Research Ethics (REK 236654). Written informed consent was obtained from all subjects. Participants received the consent form by email before the study visit to allow adequate time to read and understand the protocol and to familiarise themselves with the risk, burdens and benefits of participation in the study. In addition, one of the researchers went through the consent form in oral with the participants, and participants were explicitly allowed to ask additional questions before signing the consent form. Participants who communicated great discomfort, either orally or by body language (syncope, etc.), during the study visit were excluded from the study. Participants were also excluded if there were difficulties with blood sampling from the venous catheter (*n* 2).

## Results

### Study participants

**Figure 2.**
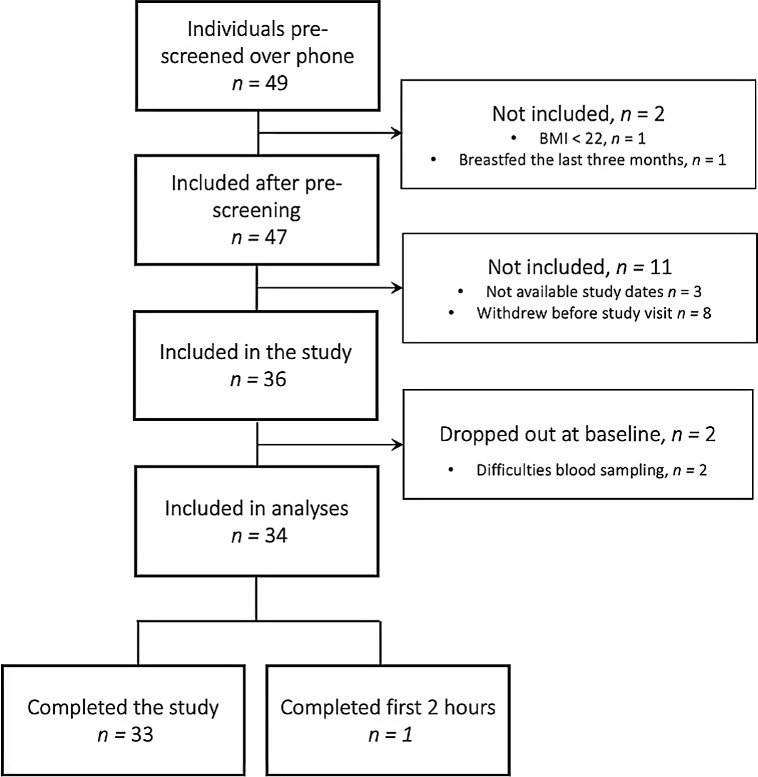
Flow chart of the inclusion process for participants in the Postprandial Metabolism Study.

A flow chart depicting the inclusion of participants is illustrated in Fig. 2. A total of forty-nine individuals completed the web-based questionnaire and were contacted by phone for a pre-screening. Of these, forty-seven individuals fulfilled the inclusion and exclusion criteria. For three of the subjects, an agreed date for participation could not be found, while eight subjects withdrew before the study visit. Therefore, a total of thirty-six individuals were included in the study. Two participants (both female) withdrew from the study right after the breakfast meal due to difficulties with blood sampling and were excluded from all analyses. Additionally, one participant completed the first 2 h (eight blood collection timepoints) before withdrawing due to difficulties with blood sampling. This participant was included in the analyses. In total, thirty-three participants completed the whole study, while data from thirty-four participants are included in the analyses.

### Participant characteristics

Complete participant characteristics are provided in Table 3. A total of eighteen males and sixteen females were included in the analyses. The age ranged from 20 to 30 years old, and thirty-three out of thirty-four participants were of Caucasian ethnicity, with one participant of Asian ethnicity. Of the female participants, thirteen participants (81 %) used some form of contraceptive, with the most common being combined oral contraceptives (*n* 6), followed by contraceptive implant (*n* 3), hormonal intra-uterine device (*n* 2), copper intra-uterine device (*n* 1) and progestin-only pill (*n* 1). On average, male participants had slightly higher BMI, waist circumference and RMR but a lower percentage of fat mass compared with female participants. All participants were considered healthy as evaluated by self-reported questionnaires and routine clinical measurements (online Supplementary Table 1).

**Table 3.** The main characteristics of the participants (*n* 34) included in the Postprandial Metabolism Study

	Total population *n* 34			Males *n* 18			Females *n* 16		
	gMean	gSD	Min–max	gMean	gSD	Min–max	gMean	gSD	Min–max
Age, years	25·3	1·13	20–30	26·1	1·12	20–30	24·5	1·13	20–29
Caucasian ethnicity									
* n*	33		–	18		–	15		–
%	97 %			100 %			93·7 %		
Height, cm	176·3	1·06	152·5–194·7	184·2	1·03	173·2–194·7	167·8	1·05	152·5–180·9
Weight, kg	73·3	1·15	57·2–98·3	80·9	1·10	64·7–98·3	65·2	1·09	57·2–76·6
BMI, kg/m^2^	23·5	1·07	202–26·9	23·8	1·07	21·2–26·9	23·1	1·07	20·2–25·5
Waist circumference, cm	81·5	1·11	67·3–100	88·1	1·08	80–100	74·6	1·06	67·3–80·0
Fat mass percentage*	22·4	1·43	10·5–41·9	18·3	1·41	10·5–39·1	28·6	1·19	21·6–41·9
RMR*	1508	1·21	1053–2200	1740	1·10	1433–2200	1269	1·12	1053–1552
Use of contraceptives									
* n*	13		–	–		–	13		–
%	38·2 %						81·3 %		

* Estimated using BodPod (COSMED).

### Change in metabolite concentrations during 24 h after the meal

The relative change in concentrations of the metabolites investigated, accompanied with the ICC, is presented in Table 4 (total population) and Table 5 (males and females separately). Further, the absolute metabolite concentrations at all timepoints are presented in online Supplementary Table 2 (total population) and online Supplementary Table 3 (males and females separately). Figures illustrating the relative change in biomarker concentrations for the total population can be found in online Supplementary Figures 2–5.

**Table 4.** The relative change in metabolite concentrations (% change from reference values) after consumption of a standardized meal in healthy subjects in the Postprandial Metabolism Study

Serum biomarker	Time after meal														ICC
	**Ref** *	15 min	30 min	45 min	60 min	90 min	2 hours	3 hours	4 hours	6 hours	8 hours	10 hours	12 hours	24 hours	
*n*	34	34	34	34	34	34	34	33	33	33	33	33	33	33	-
**Glucose and insulin**															
Glucose†	5·27 mmol/L	41·8	40·9	20·0	8·52	−0·13	−4·97	−1·58	−2·19	−6·54	−11·5	−11·0	−13·4	−14·0	0·39
Insulin	4·45 mIU/L	746	827	631	502	296	173	70·0	−12·3	−44·2	−52·4	−56·4	−54·4	−53·9	0·20
**Amino acids**															
Alanine	331 µmol/L	25·8	36·2	41·3	42·4	40·9	34·7	21·0	8·99	−5·89	−10·3	−12·5	−11·2	1·87	0·49
Arginine	85·8 µmol/L	21·2	15·3	16·8	14·4	18·1	19·3	8·41	−1·19	−4·64	−2·41	−1·94	2·22	0·07	0·51
Asparagine	66·5 µmol/L	31·4	30·6	29·1	27·6	26·2	21·9	9·45	−0·30	−5·42	−3·54	−5·47	−5·87	−6·22	0·49
Aspartic acid	17·7 µmol/L	−0·93	−0·28	−1·12	−0·32	−0·25	−0·45	2·51	2·49	1·43	3·36	4·92	3·55	1·45	0·56
Glutamic acid	43·1 µmol/L	−6·98	5·07	6·17	6·76	−5·60	−7·50	−6·51	−9·37	−14·2	−13·2	−10·9	−14·1	−15·6	0·53
Glutamine	506 µmol/L	5·49	5·32	4·78	4·97	8·44	9·62	8·41	6·99	6·18	7·30	6·93	4·15	0·45	0·51
Histidine	80·1 µmol/L	8·40	10·17	9·82	9·63	13·1	15·5	10·8	3·82	−1·11	−0·68	−0·44	0·90	5·80	0·52
Isoleucine	64·6 µmol/L	17·4	17·7	18·2	18·9	24·6	28·2	17·8	3·71	0·20	6·38	10·2	14·6	28·0	0·51
Leucine	128 µmol/L	14·6	14·7	15·2	15·8	20·6	23·8	15·8	5·20	3·82	9·83	14·1	16·6	37·2	0·50
Lysine	157 µmol/L	11·9	12·1	12·0	12·7	17·9	21·0	15·1	4·80	−2·49	−0·35	1·37	3·25	9·11	0·50
Phenylalanine	70·6 µmol/L	10·4	13·0	15·4	18·0	21·5	21·3	11·3	0·40	−3·80	1·93	3·80	4·76	5·01	0·50
Proline	179 µmol/L	30·6	38·3	45·6	51·2	54·7	55·5	41·2	24·8	7·75	0·08	−5·67	−10·59	−19·3	0·49
Threonine	132 µmol/L	15·3	15·7	14·0	13·3	13·2	11·4	4·20	−4·62	−10·1	−10·9	−12·6	−15·2	−14·5	0·51
Tryptophan	71·3 µmol/L	4·27	2·00	1·33	1·31	2·03	2·33	−4·08	−13·1	−18·2	−12·0	−8·37	−3·75	−11·0	0·50
Tyrosine	62·4 µmol/L	8·85	11·4	13·6	16·9	24·0	29·2	23·4	8·31	−9·12	−11·4	−13·2	−13·0	−8·98	0·51
Valine	250 µmol/L	6·79	8·04	7·88	7·81	10·7	13·7	11·7	6·27	2·17	1·56	1·77	2·78	14·4	0·50
**One-carbon metabolites**															
Betaine	33·0 µmol/L	10·9	15·6	18·4	20·4	25·1	24·9	14·9	10·2	3·80	3·40	−1·73	−3·81	−8·40	0·61
Choline	9·19 µmol/L	13·7	10·6	8·35	7·60	4·29	4·71	3·76	1·78	−0·09	5·31	−2·52	−7·24	−5·21	0·54
Cystathionine	0·18 µmol/L	−0·64	4·24	9·67	12·7	22·0	33·2	38·3	24·2	−7·52	−23·4	−28·2	−29·0	−24·6	0·64
Cysteine	251 µmol/L	−0·62	−2·66	−3·48	−3·60	−4·29	−4·81	−5·25	−3·56	0·36	5·13	7·61	9·59	11·6	0·50
DMG	3·45 µmol/L	1·57	2·32	2·24	5·37	11·9	11·6	10·0	4·88	−3·72	−4·36	4·48	5·70	8·16	0·82
Glycine	257 µmol/L	6·48	5·69	4·56	4·16	6·19	5·41	0·35	−2·29	−4·84	−3·79	−3·8	−4·69	−3·24	0·52
tHcy	8·16 µmol/L	−1·97	−3·33	−3·99	−3·30	−3·44	−3·00	−0·84	0·20	1·58	5·11	7·55	8·46	11·4	0·64
Methionine	29·3 µmol/L	15·7	16·7	18·7	20·8	27·0	29·2	14·0	−4·57	−14·0	−9·31	−8·05	−7·34	−4·69	0·49
MetSo	0·78 µmol/L	14·9	21·0	42·5	52·2	68·3	71·7	65·7	24·4	−0·82	−11·5	−17·5	−10·4	−15·5	0·25
Sarcosine	1·35 µmol/L	15·7	17·6	18·9	20·7	24·3	25·6	16·4	3·71	−9·10	−14·4	−18·5	−21·8	−30·0	0·69
Serine	131 µmol/L	14·1	15·6	14·5	13·8	12·7	12·1	6·42	1·07	−1·40	1·04	1·35	1·36	−0·44	0·50
**B-vitamin markers**															
Cobalamin	328 pmol/L	−1·92	−2·37	−2·65	−1·54	−2·26	−3·56	−1·36	0·09	0·53	2·45	5·13	5·73	13·5	0·55
FMN	8·07 nmol/L	−19·3	−24·9	−29·1	−30·0	−26·5	−22·3	−9·44	6·16	32·1	42·9	48·5	55·9	89·9	0·53
Folate	13·8 nmol/L	11·0	13·0	12·6	13·0	7·88	4·28	2·10	4·16	16·8	21·4	28·8	30·6	56·8	0·57
mNAM	106 nmol/L	20·7	17·7	10·4	8·83	10·2	7·99	−0·36	−0·46	5·92	6·68	−8·82	−28·0	1·37	0·59
MMA	0·14 µmol/L	1·33	0·15	1·08	2·09	3·71	5·92	4·14	−2·9	−12·9	−16·0	−18·4	−16·6	−12·0	0·63
Nicotinamide	203 nmol/L	38·0	24·1	6·40	−5·76	−13·1	−18·5	−22·9	−11·7	−8·12	−8·84	−17·1	−32·9	−11·3	0·54
Pyridoxal	17·1 nmol/L	10·8	13·1	13·7	13·0	7·64	6·78	−0·44	−3·75	−4·81	−5·61	−5·26	−5·39	−8·31	0·59
PLP	54·5 nmol/L	−3·05	−5·27	−7·62	−8·86	−10·5	−11·3	−10·9	−10·9	−12·6	−14·3	−17·4	−19·6	−14·9	0·59
4-pyridoxic acid	18·9 nmol/L	−3·08	−8·54	−18·3	−19·4	−24·6	−26·7	−24·8	−22·4	−21·6	−24·9	−24·5	−26·6	3·33	0·55
Riboflavin	13·5 nmol/L	−0·68	1·21	2·59	3·02	−0·42	−1·07	−4·39	−3·02	3·03	1·81	11·3	13·1	33·8	0·78
Thiamine	7·34 nmol/L	19·5	23·8	21·1	19·3	14·8	8·99	5·43	3·64	−1·13	−2·09	−3·11	−10·3	−19·8	0·60
TMP	3·05 nmol/L	11·4	4·79	4·65	−0·91	−3·85	−4·30	7·95	14·7	13·9	3·54	−3·05	−6·27	−12·5	0·88

ICC, Intraclass correlation coefficient; DMG, Dimethylglycine; FMN, Flavin mononucleotide; MetSo, Methionine sulfoxide; mNAM, N^1^-methyl nicotinamide; MMA, Methylmalonic acid; PLP, Pyridoxal-5´-phosphate; tHcy, Total homocysteine; TMP, Thiamine monophosphate.

* Reference values are reported as geometric means. The reference values were measured around 8 a.m., 12 hours after a standard evening meal.

† Capillary glucose.

**Table 5.** The relative change in metabolite concentrations (% change from reference values) after consumption of a standardized meal in males (*n* = 18) and females (*n* = 16) subjects in the Postprandial Metabolism Study

Serum biomarker		Time after meal														ICC
	Sex*	Ref**†**	15 min	30 min	45 min	60 min	90 min	2 hours	3 hours	4 hours	6 hours	8 hours	10 hours	12 hours	24 hours	
*n*	-	34	34	34	34	34	34	34	33	33	33	33	33	33	33	-
**Glucose and insulin**																
Glucose‡	M	5·40 mmol/L	40·4	41·6	21·5	7·00	−4·49	−6·47	−5·41	−6·00	−8·37	−13·3	−13·8	−13·3	−15·1	0·09
	F	5·12 mmol/L	43·3	40·6	18·4	10·3	5·01	−3·26	2·92	2·58	−4·30	−9·17	−7·54	−13·5	−12·6	0·45
Insulin	M	4·53 mIU/L	707	810	621	484	283	173	60·6	−25·9	−56·6	−57·3	−64·8	−62·6	−61·1	0·14
	F	4·37 mIU/L	792	846	641	523	312	173	81·8	7·4	−24·6	−45·8	−43·5	−42·2	−43·6	0·28
**Amino acids**																
Alanine	M	342 µmol/L	24·4	34·2	38·7	40·2	38·2	33·6	18·6	7·76	−6·26	−9·89	−11·0	−11·5	0·32	0·14
	F	321 µmol/L	27·4	38·5	44·3	44·9	44·0	36·0	24·1	10·5	−5·45	−10·8	−14·3	−10·8	3·75	0·50
Arginine	M	87·5 µmol/L	22·0	16·9	17·4	16·3	19·0	19·3	7·68	−1·46	−4·20	−1·96	−1·43	1·71	−0·43	0·67
	F	84·0 µmol/L	20·4	13·5	16·0	12·3	17·1	19·2	9·3	−0·87	−5·16	−2·94	−2·55	2·85	0·67	0·50
Asparagine	M	68·7 µmol/L	29·6	30·2	27·9	26·2	23·7	20·4	6·82	−2·39	−5·99	−4·44	−5·80	−6·93	−3·80	0·33
	F	64·0 µmol/L	33·5	31·1	30·5	29·1	29·1	23·7	12·7	2·26	−4·73	−2·44	−5·06	−4·58	−9·04	0·50
Aspartartic acid	M	16·1 µmol/L	−0·45	1·26	0·41	1·52	1·36	0·38	1·84	1·40	0·03	3·03	4·69	3·72	2·78	0·88
	F	19·6 µmol/L	−1·46	−1·99	−2·81	−2·36	−2·02	−1·38	3·32	3·82	3·13	3·76	5·20	3·34	−0·14	0·53
Glutamic acid	M	46·4 µmol/L	−5·16	6·76	4·10	6·17	−7·39	−9·9	−7·87	−12·7	−14·9	−16·6	−13·8	−12·0	−17·0	0·62
	F	39·6 µmol/L	−9·00	3·19	8·55	7·44	−3·54	−4·72	−4·84	−5·23	−13·4	−8·85	−7·19	−16·5	−13·9	0·51
Glutamine	M	551 µmol/L	5·45	6·03	5·68	5·51	8·48	10·3	7·51	5·47	5·97	7·32	5·82	2·44	1·53	0·73
	F	459 µmol/L	5·54	4·54	3·78	4·37	8·40	8·89	9·50	8·85	6·43	7·27	8·29	6·24	−0·82	0·50
Histidine	M	79·4 µmol/L	7·74	10·6	10·7	10·1	12·8	15·7	9·47	2·38	−1·18	−0·61	−0·61	0·48	7·32	0·92
	F	80·8 µmol/L	9·15	9·71	8·84	9·16	13·4	15·2	12·4	5·58	−1·02	−0·75	−0·24	1·41	4·00	0·50
Isoleucine	M	68·5 µmol/L	18·0	19·1	19·1	19·6	21·8	23·2	9·36	−4·24	−4·17	3·09	6·25	10·2	21·1	0·41
	F	60·4 µmol/L	16·6	16·0	17·2	18·2	27·7	34·1	28·7	14·1	5·70	10·5	15·2	20·0	36·7	0·50
Leucine	M	136 µmol/L	15·1	15·6	16·1	16·5	18·6	20·0	9·22	−0·97	0·94	7·75	11·0	13·4	33·4	0·40
	F	120 µmol/L	14·1	13·7	14·2	15·0	22·8	28·2	24·2	13·1	7·40	12·4	17·9	20·6	41·9	0·50
Lysine	M	162 µmol/L	12·4	13·3	14·0	14·7	18·4	21·3	13·9	3·81	−1·81	0·57	2·10	3·99	11·4	0·42
	F	151 µmol/L	11·3	10·8	9·91	10·4	17·4	20·7	16·5	6·00	−3·30	−1·44	0·51	2·35	6·42	0·50
Phenylalanine	M	70·3 µmol/L	10·7	13·1	15·2	17·1	19·1	17·8	5·16	−4·63	−5·37	1·52	3·88	4·84	4·89	0·33
	F	71·0 µmol/L	10·1	12·8	15·6	19·0	24·3	25·4	19·0	6·78	−1·88	2·43	3·71	4·67	5·16	0·50
Proline	M	203 µmol/L	27·2	33·7	38·8	42·7	44·1	46·2	31·5	18·0	5·64	−0·09	−5·08	−9·49	−18·3	0·33
	F	155 µmol/L	34·6	43·7	53·7	61·3	67·6	66·8	53·8	33·4	10·3	0·30	−6·37	−11·9	−20·5	0·49
Threonine	M	140 µmol/L	13·5	15·0	14·8	12·7	11·1	10·2	2·31	−6·53	−10·2	−11·3	−12·9	−16·0	−13·7	0·37
	F	125 µmol/L	17·4	16·6	13·1	13·9	15·6	12·7	6·50	−2·28	−10·1	−10·4	−12·3	−14·2	−15·3	0·51
Tryptophan	M	75·1 µmol/L	4·48	2·02	1·26	1·18	0·98	0·56	−6·94	−14·6	−18·4	−13·4	−7·70	−3·90	−12·0	0·47
	F	67·3 µmol/L	4·04	1·98	1·40	1·46	3·22	4·36	−0·53	−11·1	−17·9	−10·4	−9·16	−3·56	−9·71	0·50
Tyrosine	M	65·6 µmol/L	9·51	12·4	14·5	17·3	22·3	26·1	17·1	3·40	−9·60	−11·3	−13·0	−12·9	−10·6	0·39
	F	58·9 µmol/L	8·12	10·2	12·6	16·4	26·0	32·8	31·5	14·5	−8·54	−11·6	−13·6	−13·2	−6·95	0·51
Valine	M	259 µmol/L	7·07	8·61	8·96	8·80	10·5	12·7	8·46	2·52	−0·01	−0·01	0·27	1·22	12·4	0·68
	F	241 µmol/L	6·48	7·41	6·69	6·71	11·0	14·8	15·8	11·0	4·86	3·47	3·61	4·67	17·0	0·50
**One-carbon metabolites**																
Betaine	M	37·6 µmol/L	11·1	16·9	19·9	20·6	23·2	23·2	14·7	10·7	2·29	2·16	−2·50	−3·60	−3·12	0·82
	F	28·6 µmol/L	10·6	14·1	16·7	20·1	27·2	26·9	15·1	9·44	5·64	4·90	−0·81	−4·06	−14·4	0·59
Choline	M	9·34 µmol/L	12·7	10·3	7·70	8·03	3·27	3·84	2·91	3·33	0·92	6·74	−0·90	−1·79	−2·90	0·74
	F	9·04 µmol/L	14·8	10·9	9·03	7·12	5·46	5·69	4·80	−0·06	−1·28	3·61	−4·43	−13·4	−7·90	0·51
Cystathionine	M	0·18 µmol/L	−0·79	3·16	9·83	14·8	23·9	34·9	38·5	22·4	−6·81	−20·8	−25·5	−25·5	−22·3	0·75
	F	0·17 µmol/L	−0·46	5·46	9·49	10·4	19·8	31·3	38·2	26·4	−8·38	−26·3	−31·2	−32·9	−27·4	0·54
Cysteine	M	257 µmol/L	−1·03	−2·80	−4·04	−4·31	−4·59	−4·62	−5·14	−3·51	0·44	5·00	7·46	9·70	11·9	0·71
	F	244 µmol/L	−0·15	−2·51	−2·84	−2·80	−3·95	−5·03	−5·38	−3·63	0·25	5·28	7·78	9·45	11·3	0·50
DMG	M	3·66 µmol/L	6·37	3·81	2·50	8·09	15·5	13·9	13·3	6·98	−2·16	−4·82	4·64	5·49	6·92	0·84
	F	3·22 µmol/L	−3·58	0·66	1·94	2·40	8·03	9·11	6·21	2·43	−5·56	−3·81	4·30	5·96	9·67	0·78
Glycine	M	260 µmol/L	7·33	7·56	6·96	6·41	8·07	7·88	2·18	−0·85	−3·18	−2·71	−2·77	−4·44	−1·40	0·67
	F	253 µmol/L	5·54	3·63	1·92	1·68	4·12	2·70	−1·79	−4·00	−6·80	−5·07	−5·02	−5·00	−5·41	0·52
tHcy	M	8·69 µmol/L	−1·62	−3·08	−4·28	−3·55	−2·76	−1·83	−0·12	0·71	2·43	6·75	8·66	10·2	13·1	0·93
	F	7·60 µmol/L	−2·37	−3·61	−3·67	−3·02	−4·21	−4·31	−1·70	−0·41	0·56	3·17	6·23	6·37	9·34	0·58
Methionine	M	30·7 µmol/L	16·3	17·9	19·5	21·4	26·3	27·3	8·66	−8·70	−14·4	−9·58	−7·81	−7·31	−5·07	0·27
	F	27·8 µmol/L	15·0	15·4	17·7	20·2	27·8	31·3	20·8	0·62	−13·5	−8·99	−8·34	−7·37	−4·24	0·50
MetSo	M	0·74 µmol/L	7·50	17·2	34·3	43·8	52·5	67·3	49·9	11·6	−0·43	−17·5	−14·3	−6·70	−19·4	0·16
	F	0·82 µmol/L	23·8	25·4	52·2	66·8	88·1	76·9	86·7	41·6	−1·30	−3·64	−21·2	−14·5	−10·5	0·23
Sarcosine	M	1·52 µmol/L	16·3	18·7	19·6	20·7	22·6	23·6	12·9	1·97	−8·51	−14·1	−17·8	−21·1	−29·1	0·58
	F	1·18 µmol/L	15·0	16·4	18·1	20·7	26·4	27·8	20·7	5·83	−9·82	−14·7	−19·3	−22·6	−31·1	0·69
Serine	M	129 µmol/L	14·0	16·2	15·5	14·3	12·2	11·7	4·13	−0·74	−2·74	−1·07	−0·42	−0·11	0·38	0·26
	F	133 µmol/L	14·3	14·8	13·4	13·3	13·4	12·5	9·25	3·28	0·24	3·62	3·51	3·16	−1·42	0·50
**B-vitamin markers**																
Cobalamin	M	330 pmol/L	−2·45	−3·50	−5·04	−2·33	−3·33	−5·62	−1·77	0·81	0·45	1·83	4·75	5·28	12·9	0·97
	F	325 pmol/L	−1·31	−1·08	0·11	−0·64	−1·03	−1·17	−0·86	−0·75	0·63	3·20	5·58	6·27	14·3	0·52
FMN	M	7·75 nmol/L	−23·1	−28·1	−28·3	−31·6	−29·4	−24·9	−14·0	1·42	28·1	46·7	44·2	58·9	92·1	0·43
	F	8·44 nmol/L	−14·8	−21·2	−30·0	−28·2	−23·0	−19·3	−3·70	12·2	37·0	38·4	53·9	52·3	87·4	0·54
Folate	M	14·0 nmol/L	12·6	14·3	14·7	13·6	7·89	3·68	3·94	5·22	19·9	28·6	29·0	27·8	50·2	0·63
	F	13·6 nmol/L	10·1	12·0	11·6	11·8	8·16	4·77	0·14	3·71	13·5	17·0	28·9	33·5	65·4	0·54
mNAM	M	90·6 nmol/L	17·6	16·2	10·7	7·55	13·8	16·6	6·79	12·6	20·9	20·3	3·47	−21·9	6·58	0·73
	F	126 nmol/L	24·3	19·4	9·99	10·3	6·38	−0·95	−8·31	−14·1	−9·59	−7·60	−21·7	−34·7	−4·54	0·52
MMA	M	0·13 µmol/L	0·11	−0·30	1·24	3·47	5·24	7·02	5·52	−2·56	−12·0	−14·6	−15·6	−15·9	−14·1	0·71
	F	0·14 µmol/L	2·72	0·65	0·89	0·56	2·01	4·70	2·51	−3·32	−14·0	−17·7	−21·6	−17·3	−9·33	0·61
Nicotinamide	M	194 nmol/L	52·9	45·8	32·8	13·6	5·94	−2·50	−4·82	7·25	14·3	5·74	−4·30	−30·0	−11·4	0·60
	F	214 nmol/L	23·8	3·70	−14·9	−23·7	−31·1	−34·8	−39·4	−27·5	−25·4	−23·6	−29·5	−36·6	−10·2	0·52
Pyridoxal	M	18·9 nmol/L	9·08	10·6	11·0	8·92	4·21	3·30	−5·09	−4·60	−6·69	−8·19	−6·98	−7·51	−8·71	0·87
	F	15·2 nmol/L	12·7	16·1	16·8	17·8	11·6	10·8	5·44	−2·71	−2·52	−2·42	−3·14	−2·79	−7·83	0·54
PLP	M	61·9 nmol/L	−3·04	−4·85	−7·50	−8·39	−11·2	−11·1	−12·5	−11·9	−13·1	−14·4	−16·5	−17·8	−13·0	0·95
	F	47·2 nmol/L	−3·06	−5·74	−7·75	−9·39	−9·55	−11·5	−8·85	−9·7	−11·9	−14·2	−18·3	−21·7	−17·2	0·53
4-pyridoxic acid	M	19·6 nmol/L	−4·03	−7·59	−17·4	−18·0	−22·8	−24·6	−22·9	−17·6	−16·2	−22·9	−21·6	−25·3	2·97	0·68
	F	18·2 nmol/L	−2·01	−9·60	−19·2	−20·9	−26·6	−29·0	−26·9	−27·8	−27·6	−27·3	−27·8	−28·2	3·77	0·52
Riboflavin	M	11·2 nmol/L	−1·23	1·50	3·10	1·99	1·17	−3·56	−5·57	−6·46	1·51	−0·71	8·52	13·4	29·4	0·94
	F	16·5 nmol/L	−0·06	0·89	2·02	4·20	−2·19	1·80	−2·94	1·28	4·87	4·92	14·7	12·6	39·4	0·64
Thiamine	M	7·01 nmol/L	21·6	24·5	21·8	20·1	15·5	9·87	2·24	1·64	−0·81	−2·75	−1·43	−7·24	−17·8	0·69
	F	7·73 nmol/L	17·3	23·0	20·3	18·3	14·1	8·01	9·38	6·09	−1·52	−1·29	−5·08	−13·8	−22·2	0·56
TMP	M	3·04 nmol/L	12·9	7·66	5·97	1·26	2·26	2·09	11·2	19·3	17·5	12·8	0·61	5·92	−7·79	0·79
	F	3·05 nmol/L	9·65	1·65	3·19	−3·30	−10·3	−11·0	4·15	9·35	9·65	−6·52	−7·26	−19·1	−17·9	0·92

ICC, Intraclass correlation coefficient; DMG, Dimethylglycine; FMN, Flavin mononucleotide; MetSo, Methionine sulfoxide; mNAM, N^1^-methyl nicotinamide; MMA, Methylmalonic acid; PLP, Pyridoxal-5´-phosphate; tHcy, Total homocysteine; TMP, Thiamine monophosphate.

* Male/Female.

† Reference values are reported as geometric means. The reference values were measured around 8 a.m., 12 hours after a standard evening meal.

‡ Capillary glucose.

### Glucose and insulin

**Figure 3.**
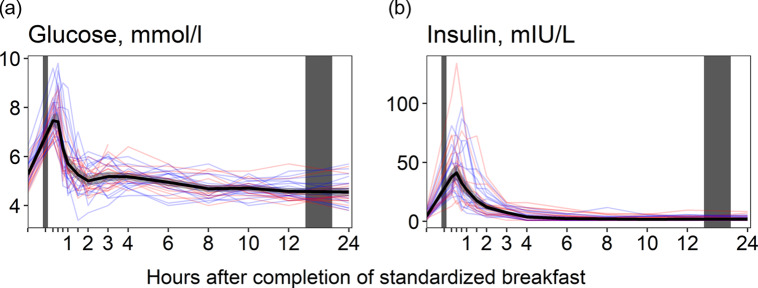
The concentrations of glucose and insulin as a function of time since completion of the standardised breakfast meal in participants in the Postprandial Metabolism Study (*n* = 34). The solid black line represents the geometric mean, while the grey shaded area represents the 95% geometric confidence intervals. The blue and red lines represent the male and female participants, respectively. The leftmost vertical line indicates the time of the standardised breakfast meal, while the rightmost vertical line indicates time spent outside the study centre.

Glucose concentrations (Fig. 3(a)) increased immediately after the meal, peaking at 15 min (+41·8 % increase), before returning to baseline values at 90 min after the meal. Concentrations decreased slightly thereafter, falling to their lowest values at 24 h (–14·0 % decrease from baseline levels). For insulin (Fig. 3(b)), we observed a similar pattern, with concentrations peaking at 30 min (+827 % increase) and thereafter decreasing, falling to their lowest values at 10 h (–56·4 % decrease from baseline values). The results were comparable between the sexes (Table 5).

### Amino acids

**Figure 4.**
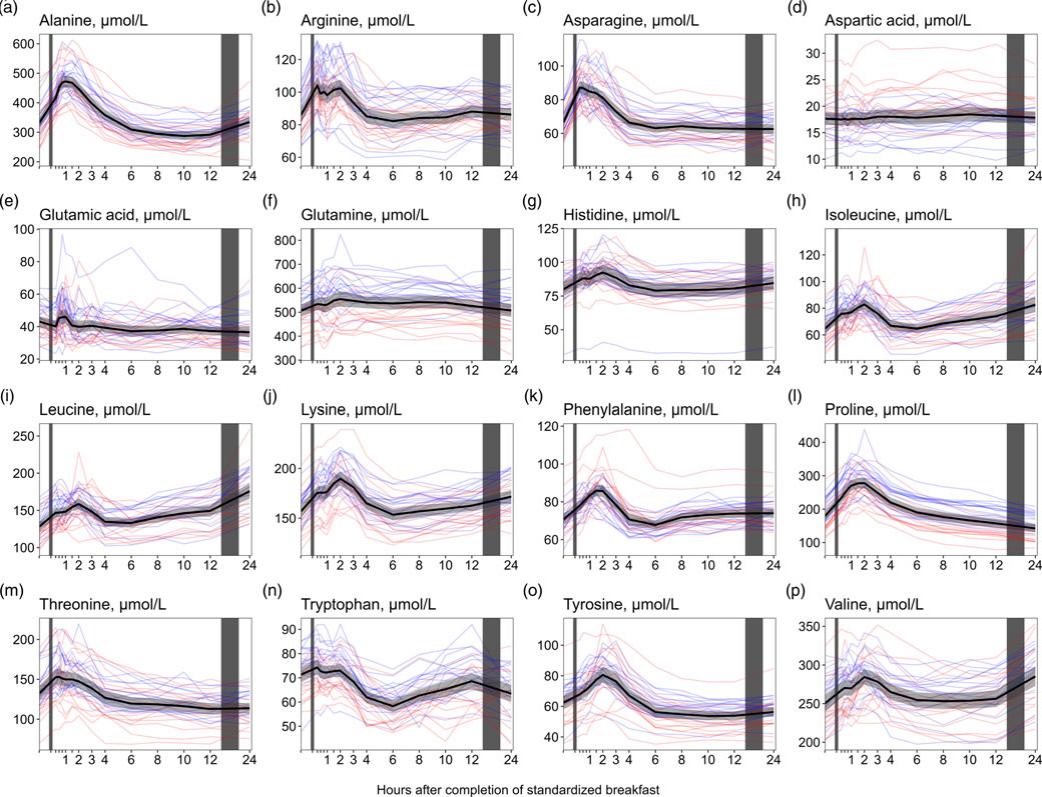
The concentrations of amino acids as a function of time since completion of the standardised breakfast meal in participants in the Postprandial Metabolism Study (*n* = 34). The solid black line represents the geometric mean, while the grey shaded area represents the 95% geometric confidence intervals. The blue and red lines represent the male and female participants, respectively. The leftmost vertical line indicates the time of the standardised breakfast meal, while the rightmost vertical line indicates time spent outside the study centre.

We observed a consistent pattern for nearly all amino acids, with increased concentrations right after the meal. This pattern was observed for alanine, arginine, asparagine, histidine, isoleucine, leucine, lysine, phenylalanine, proline, threonine, tyrosine and valine (Fig. 4(a)–(p)). The largest relative increases in serum concentrations were observed for proline (Fig. 4(l), +55·5 % increase from baseline values), alanine (Fig. 4(a), +42·4 %) and tyrosine (Fig. 4(o), +29·2 %). The concentrations of the BCAA (Fig. 4(h), (i), (p)) increased from 6 (isoleucine and leucine) or 8 (valine) h to 24 h, with peak concentrations observed at 24 h after the meal (isoleucine: +28·0 %, leucine: +37·2 %, valine: +14·4 % increase from baseline values). In contrast, the concentrations of proline (Fig. 4(l)) decreased from the 6- to the 24-h timepoint, reaching the lowest values at 24 h (–19·3 % decrease from baseline values). The levels of aspartic acid (Fig. 4(d)) fluctuated immediately after the meal but stabilised around baseline levels at 3 h, while the levels of glutamic acid (Fig. 4(e)) fluctuated during the first hour after the meal before remaining decreased from 90 min onwards, with the lowest values observed at 24 h (–15·6 % decrease from baseline values). For glutamine (Fig. 4(f)), the concentrations appeared to be slightly elevated during the first 12 h, before returning to baseline values 24 h after the meal. The ICC for the amino acids ranged from 0·49 to 0·56. Male participants had, on average, slightly higher concentrations of nearly all amino acids except for aspartic acid, in which females had higher concentrations. However, the relative changes in concentrations after the meal were comparable between sexes for all amino acids (Table 5).

### One-carbon metabolites

**Figure 5.**
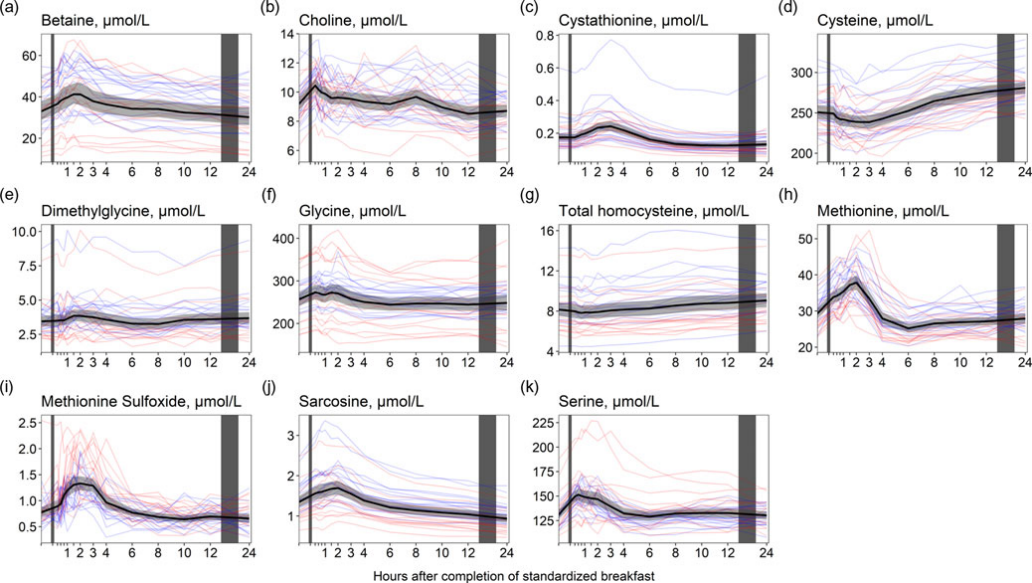
The concentrations of one-carbon metabolites as a function of time since completion of the standardised breakfast meal in participants in the Postprandial Metabolism Study (*n* = 34). The solid black line represents the geometric mean, while the grey shaded area represents the 95% geometric confidence intervals. The blue and red lines represent the male and female participants, respectively. The leftmost vertical line indicates the time of the standardised breakfast meal, while the rightmost vertical line indicates time spent outside the study centre.

For the one-carbon metabolites, we observed similar results to those observed for the amino acids; the concentrations of betaine, choline, dimethylglycine and the amino acids cystathionine, glycine, methionine, sarcosine and serine increased and peaked within the first 3 h after completion of the meal (Fig. 5). The largest relative increase was observed for cystathionine (Fig. 5(c), +38·3 % increase from baseline values), methionine (Fig. 5(h), +29·2 %) and betaine (Fig. 5(a), +25·1 %). The concentrations were thereafter relatively stable until 24 h after the meal, except for cystathionine, which decreased to its lowest values at 12 h (–29·0% decrease from baseline values). For cysteine and homocysteine (Fig. 5(d) and (g)), the opposite was observed, with slightly decreased concentrations immediately after the meal followed by increased concentrations peaking at 24 h (+11·6% and 11·4% increase, respectively). The ICC for the one-carbon metabolites ranged from 0·25 (methionine sulfoxide) to 0·82 (dimethylglycine). As with the amino acids, the males had on average slightly higher concentrations of all one-carbon metabolites except for methionine sulfoxide and serine in which the females had slightly higher concentrations. Further, we observed that the females had a higher peak in concentrations of methionine sulfoxide than males (+88·1 % at 90 minutes after the meal for females; +67·3 % at 2 h for males). The relative changes for the other one-carbon metabolites were comparable between the sexes (Table 5).

### B-vitamin biomarkers

**Figure 6.**
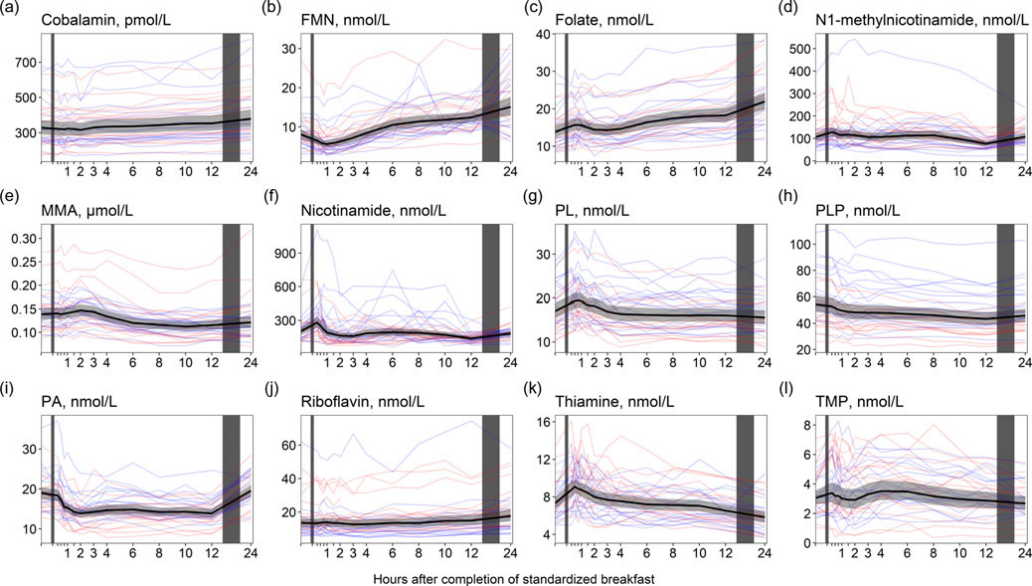
The concentrations of B-vitamin biomarkers as a function of time since completion of the standardised breakfast meal in participants in the Postprandial Metabolism Study (*n* = 34). The solid black line represents the geometric mean, while the grey shaded area represents the 95% geometric confidence intervals. The blue and red lines represent the male and female participants, respectively. The leftmost vertical line indicates the time of the standardised breakfast meal, while the rightmost vertical line indicates time spent outside the study centre.

For cobalamin and folate (Fig. 6(a) and (c)), we observed no considerable changes in concentrations immediately after the meal, except for a small peak in folate concentrations in the first hour after the meal (+13·0% increase). However, the concentrations of both cobalamin and folate steadily increased from 3 h, peaking at 24 h after the meal (+13·5 % increase for cobalamin and +56·8 % increase for folate). A similar pattern was observed for FMN and riboflavin (Fig. 6(b) and (j)), with the highest concentrations at 24 h (+89·9 % and +33·8 % increase for FMN and riboflavin, respectively). For the vitamin B_6_ vitamers (Fig. 6(g)–(i)), a slight increase in pyridoxal concentrations was observed right after the meal, peaking at 45 min (+13·7 % increase), with concentrations returning to baseline at 3 h and thereafter slightly decreasing until 24 h. Pyridoxal-5-phosphate concentrations decreased in the hours after the meal, with the lowest values observed at 12 h (–19·6 % decrease). The concentration of 4´-pyridoxic acid decreased right after the meal with the lowest values observed at 2 h (–26·6 % decrease) and remained decreased until 12 h but thereafter increased and returned to baseline values at 24 h. The concentrations of N1´-methylnicotinamide, thiamine and TMP (Fig. 6(d), (k), (l)) increased right after the meal, peaking within the first hour (N1´-methylnicotinamide: +20·7 % increase, thiamine: +23·8% increase, TMP: +11·4 % increase). The concentrations thereafter decreased, with N1´-methylnicotinamide reaching the lowest values at 12 h (–28·0 % decrease from baseline values) and thiamine and TMP reaching the lowest values at 24 h (thiamine: –19·8 % decrease, TMP: –12·5 %). The concentrations of methylmalonic acid (Fig. 6(e)) slightly increased the first 3 h after the meal but thereafter decreased, reaching the lowest levels at 10 h (–18·4 % decrease from baseline values). The ICC for the B-vitamin biomarkers ranged from 0·53 (FMN) to 0·88 (TMP). The results were largely similar between the sexes, except for the change in concentrations of N1´-methylnicotinamide and nicotinamide (Table 5). For N1´-methylnicotinamide, we observed that concentrations immediately increased at 15 min in females but thereafter steadily decreased until 12 h after the meal. In males, the concentrations increased and remained elevated until 8 h after the meal before decreasing. For nicotinamide, the concentrations slightly increased the first hour after the meal in females, and thereafter decreased and remained lowered until 12 h after the meal. In males, the concentrations increased and remained elevated or at baseline levels the first 8 h after the meal before thereafter decreasing.

## Discussion

In this study of thirty-four healthy participants, we observed changes in circulating concentrations of a number of serum biomarkers and metabolites after the consumption of a standardised breakfast meal followed by food abstinence for 24 h.

### Potential mechanisms and comparison with other studies

#### Amino acids

Consistent with previous studies, we observed that males generally exhibited higher concentrations of most serum amino acids compared with females^(23,24)^. However, the relative changes in amino acids were comparable between sexes. Further, our findings on amino acids are consistent with several previous studies. In a review by LaBarre and colleagues^(25)^, they reported that the blood concentrations of amino acids tended to peak at 60–90 min after a mixed macronutrient challenge and return to baseline values 4 h after the meal. However, while most of the studies included in the LaBarre review examined the effect of specific foods or comparisons like cod *v*. beef, or rye bread *v*. wheat bread, our study demonstrates that amino acid concentrations follow this pattern even after a regular breakfast meal. The amino acid profile probably reflects the digestion and absorption of proteins, wherein proteins are cleaved into dipeptides, tripeptides and amino acids^(26)^. Yet, the meal amino acid composition might not fully predict the postprandial blood response. Similar to the present study, Badoud *et al.* ^(27)^ reported the largest relative increase in concentrations postprandially for proline and alanine after a high-energy breakfast. Intriguingly, the most abundant amino acids in the high-energy breakfast were leucine, glutamic acid and proline, indicating that the amino acid postprandial response cannot entirely be explained by the abundance of the amino acids eaten. Further, glutamic acid is one of the most abundant amino acids found in dietary protein^(28)^, but in the present study, we observed only small changes in glutamic acid after the meal compared with most other amino acids, which has also been observed by others^(29)^. It has been suggested that glutamic acid is metabolised to various amino acids in the enterocyte, mainly alanine but also proline^(28)^, which may explain the observations in the present study. Similarly to our observations at 24 h, Krüg *et al.* ^(5)^ and Rubio-Aliaga *et al.* ^(4)^ reported increased concentrations of the BCAA during 36 h of fasting. The BCAA cannot be synthesised *de novo* and must be obtained from the diet or by proteolysis. During fasting, the main source of BCAA in the blood is protein degradation^(30)^, predominantly derived from skeletal muscle^(31)^. It has been suggested that increased serum concentrations of BCAA during fasting are related to decreased glycolysis and increased fatty acid oxidation and proteolysis in muscles^(32)^.

#### One-carbon metabolites

Among the one-carbon metabolites, serum concentrations of cystathionine and methionine were most responsive to food intake and subsequent fasting. It is well known that methionine levels increase and peak 1 h after a methionine loading test^(33)^. Although the methionine content of the breakfast meal in the present study was lower than that typically ingested in a methionine loading test, the breakfast meal (including cheese and whole grains) likely caused the observed increase in methionine, which peaked at 2 h after the meal. Methionine can be converted to homocysteine through the methionine–homocysteine cycle, which may further be converted to cystathionine in the transsulphuration pathway^(34)^. It has previously been shown that cystathionine concentrations increase after the intake of methionine^(35)^. In the present study, the temporal pattern is consistent with methionine being released from protein in the food, followed by an increase in cystathionine, peaking at 3 h after the meal. We also observed increased concentrations of betaine, choline, dimethylglycine, glycine and sarcosine in the first hours after the meal. All these metabolites are involved in the choline oxidation pathway. Calculations using data from the USDA food database^(36)^ suggest that the betaine and choline content in the breakfast was approximately 113 and 25 mg, respectively, which may explain the increased concentrations of the metabolites in the choline oxidation pathway. Interestingly, we observed slightly decreased concentrations of homocysteine and cysteine after the meal, contrary to the findings for the other one-carbon metabolites. This has been reported previously at 1 and 2 h after a meal^(18)^ and may have several possible explanations. First, increased availability of choline and betaine may facilitate the remethylation of homocysteine to methionine using betaine or 5-methyltetrahydrofolate as a methyl donor. Second, the decreased concentrations of homocysteine and cysteine, accompanied by increased concentrations of cystathionine immediately after the meal, may indicate an increased conversion of homocysteine to cystathionine through the transsulfuration pathway.

#### B-vitamin biomarkers

In this study, we observed a sharp increase in thiamine and TMP concentrations immediately after the meal, consistent with our previous findings using cross-sectional data^(37)^. Both free thiamine and TMP enter the bloodstream during the absorption of thiamine^(38)^, and the observed peak might be attributable to the thiamine content from the meal, which is estimated to be about 0·54 mg (Table 2). Further, we observed a sharp decrease in FMN concentrations, reaching the lowest value (–30·0 % decrease) at 1 h after the meal, similar to what has been reported previously^(18,37,39)^. FMN serves as a cofactor in the electron transport chain, and the sharp decrease may indicate increased utilisation as a cofactor in the first hour after a meal. We observed increases in FMN and riboflavin concentrations from 4 to 24 h. To our knowledge, no studies have previously reported changes in FMN or riboflavin concentrations in the fasting state. As the increase in both FMN and riboflavin concentrations started around 4 h after the meal, it is unlikely the increase was due to the riboflavin content of the meal. We observed a similar pattern for folate, which was 56·8 % higher at 24 h compared with baseline values. Similar observations have been reported previously, with a doubling in folate concentrations following 36 h of fasting compared with immediately after a meal. It has been suggested that this increase may be explained by reduced excretion of folate in bile during fasting^(40)^.

### Implications

We have demonstrated that several metabolites and biomarkers change dynamically after a habitual meal in healthy, young adults. This could have implications in the clinic, where specific cut-offs in circulating biomarker concentrations are used to diagnose a disease or condition or to monitor or initiate treatment. For example, we observed that folate concentrations increased on average by 56·8 % from baseline values to 24 h. A total of six participants (17 %) had folate concentrations below the established cut-off at 10 nmol/l set by the WHO^(41)^ at baseline or during the first hours after the meal. However, at later timepoints, all these subjects had folate concentrations above this cut-off, meaning in a clinical setting they would have been classified as folate deficient if their blood sample was taken before or during the first hours after the meal. Our findings indicate that clinically it is important to accurately account for prandial status and time since last meal when evaluating certain serum biomarkers. This can be done by standardising the blood sampling timepoint, by utilising data on the dynamics of postprandial metabolism or by applying different cut-offs according to the time since food intake. Further, our findings could have implications for observational research where the time of blood sampling is rarely standardised to account for dietary intake and time since last meal. Not standardising the time of blood sampling but only distinguishing between ‘fasting’ and ‘non-fasting’ states when investigating metabolite concentrations in research may introduce measurement error. When the metabolite concentration is modelled as the independent variable, non-differential measurement error is, on average, expected to attenuate the observed associations due to regression dilution bias^(42)^. However, attenuation cannot be automatically assumed in individual studies, as the measurement error may be unequally distributed by chance^(43)^, or if the concentration is grouped into categories, such as quantile groups^(42)^. Further, if the biomarker is modelled as a confounder, non-differential measurement error may result in residual confounding, biasing the association in the same direction as the original confounding^(42)^. In this context, non-differential measurement error may result from blood samples collected at random timepoints after food intake. Differential measurement error of the biomarker, potentially arising from systematically collecting the blood samples at timepoints associated with peak or through metabolite concentration, could bias the risk association in any direction and give rise to wrong conclusions from the study^(10)^. We suggest that in future epidemiological studies, blood sampling in relation to time since last meal should be standardised to limit the impact of prandial status on circulating biomarker concentrations. When the biomarker concentration is the outcome of interest, it is crucial that sampling procedures are comparable across participants and across timepoints when repeated measurements are taken within the same individual.

### Strengths and limitations

This study has several strengths. First, we obtained data on a wide range of metabolites from thirty-four participants, including both males and females. Similar studies have usually had fewer participants^(4,6)^ or only included male subjects^(5)^. In addition, to increase the internal validity of the study, we recruited a homogenous group of participants, reducing potential variability in metabolite concentrations linked to age, health status and body composition and all participants remained inactive during the study visit. Unfortunately, this reduces the generalisability of our findings. Results for glucose and insulin were highly consistent with the *a priori* expectations^(4,5)^ and can be used as a compliance measure. Despite the homogeneity of the cohort, there were large inter-individual differences in RMR (ranging from 1053 to 2200 kcal/d) and body composition (fat mass percentage ranging from 10·5 to 41·2) between the participants. It is well known that body composition may affect postprandial responses^(44)^; thus, it is likely that the meal was metabolised at different rates, which introduces a source of variability in the results.

### Conclusion

We observed that the circulating concentration of several metabolites changed considerably after the consumption of a standardised breakfast meal. The changes were not limited only to the hours immediately after the meal, with several metabolites changing considerably during fasting for 24 h. Our findings challenge the current, imprecise, practice of distinguishing between fasting and non-fasting blood samples and have implications for using metabolites in clinical practice and in research.

## Supporting information

Anfinsen et al. supplementary material Anfinsen et al. supplementary material

## Supplementary material

For supplementary material referred to in this article, please visit https://doi.org/10.1017/S0007114523002490
