# Supplementary material for: Time-resolved concentrations of serum amino acids, one-carbon metabolites and B-vitamin biomarkers during the postprandial and fasting state: the Postprandial Metabolism in Healthy Young Adults (PoMet) Study
Source: Br J Nutr. 2023 Oct 27;131(5):786–800. doi: 10.1017/S0007114523002490 (PMC10864995; doi:10.1017/S0007114523002490)
Supplement: Anfinsen et al. supplementary material [file S0007114523002490sup001.pdf]

Time-Resolved Metabolite Concentrations during the Postprandial and Fasting State: The Postprandial Metabolism in Healthy Young Adults (PoMet) study. *Åslaug Matre Anfinsen*

Supplementary Table 1. An overview of all metabolites, analytical method, and the minimum, maximum, and mean baseline concentrations

	Laboratory (platform)[1]	Method	gMean (gSD)	Mean (SD)	Minimum	Maximum
**Amino acids**						
Serum alanine, µmol/L	Bevital (B)	GC-MS/MS	331 (1.18)	336 (57.5)	243	484
Serum arginine, µmol/L	Bevital (C)	LC-MS/MS	85.8 (1.15)	86.6 (12.0)	66.8	107
Serum asparagine, µmol/L	Bevital (B)	GC-MS/MS	66.5 (1.14)	67.0 (8.77)	53.7	84.9
Serum aspartate, µmol/L	Bevital (B)	GC-MS/MS	17.7 (1.22)	18.0 (3.54)	9.96	28.5
Serum glutamate, µmol/L	Bevital (B)	GC-MS/MS	43.1 (1.25)	44.1 (9.44)	26.9	63.6
Serum glutamine, µmol/L	Bevital (B)	GC-MS/MS	506 (1.16)	511 (70.9)	350	632
Serum histidine, µmol/L	Bevital (B)	GC-MS/MS	80.1 (1.21)	81.3 (12.0)	31.5	101
Serum isoleucine, µmol/L	Bevital (B)	GC-MS/MS	63.6 (1.20)	65.6 (11.9)	45.1	90.1
Serum leucine, µmol/L	Bevital (B)	GC-MS/MS	128 (1.16)	129 (18.8)	97.1	167
Serum lysine, µmol/L	Bevital (B)	GC-MS/MS	157 (1.13)	158 (18.6)	118	203
Serum phenylalanine, µmol/L	Bevital (B)	GC-MS/MS	70.6 (1.12)	71.1 (8.29)	54.7	99.0
Serum proline, µmol/L	Bevital (B)	GC-MS/MS	179 (1.27)	184 (43.3)	112	290
Serum threonine, µmol/L	Bevital (B)	GC-MS/MS	132 (1.23)	135 (27.1)	69.8	189
Serum tryptophan, µmol/L	Bevital (B)	GC-MS/MS	71.3 (1.14)	71.9 (8.83)	47.4	85.3
Serum tyrosine, µmol/L	Bevital (B)	GC-MS/MS	62.4 (1.23)	63.6 (12.3)	35.7	99.2
Serum valine, µmol/L	Bevital (B)	GC-MS/MS	250 (1.14)	252 (32.6)	202	325
**One-carbon metabolites**						
Serum betaine, µmol/L	Bevital (C)	LC-MS/MS	33.0 (1.49)	35.3 (11.6)	11.3	54.7
Serum choline, µmol/L	Bevital (C)	LC-MS/MS	9.19 (1.16)	9.29 (1.38)	6.35	12.9
Serum cystathionine, µmol/L	Bevital (B)	GC-MS/MS	0.18 (1.46)	0.19 (0.09)	0.11	0.60
Serum cysteine, µmol/L	Bevital (B)	GC-MS/MS	251 (1.11)	252 (25.8)	209	316

	Laboratory (platform)[1]	Method	gMean (gSD)	Mean (SD)	Minimum	Maximum
Serum DMG, µmol/L	Bevital (C)	LC-MS/MS	3.45 (1.38)	3.65 (1.42)	2.23	8.72
Serum glycine, µmol/L	Bevital (B)	GC-MS/MS	257 (1.20)	261 (47.4)	173	352
Serum total homocysteine, µmol/L	Bevital (B)	GC-MS/MS	8.16 (1.28)	8.40 (2.12)	4.53	14.3
Serum methionine, µmol/L	Bevital (B)	GC-MS/MS	29.3 (1.16)	29.6 (4.44)	22.8	38.9
Serum methionine sulfoxide, µmol/L	Bevital (C)	LC-MS/MS	0.78 (1.40)	0.83 (0.37)	0.14	2.54
Serum sarcosine, µmol/L	Bevital (B)	GC-MS/MS	1.35 (1.36)	1.41 (0.44)	0.65	2.48
Serum serine, µmol/L	Bevital (B)	GC-MS/MS	131 (1.12)	132 (15.8)	110	188
**B-vitamin markers**						
Serum cobalamin, pmol/L	MBF	Immunoassay	328 (1.43)	350 (134)	176	675
Serum FMN, nmol/L	Bevital (D)	LC-MS/MS	8.07 (1.56)	8.91 (4.51)	3.07	27.1
Serum folate, nmol/L	MBF	Immunoassay	13.8 (1.40)	14.6 (5.30)	8.40	29.7
Serum N1-methylnicotinamide, nmol/L	Bevital (D)	LC-MS/MS	106 (1.60)	120 (70.4)	44.6	388
Serum MMA, µmol/L	Bevital (B)	GC-MS/MS	0.14 (1.30)	0.14 (0.04)	0.09	0.27
Serum nicotinamide, nmol/L	Bevital (D)	LC-MS/MS	203 (1.59)	229 (133)	109	668
Serum pyridoxal, nmol/L	Bevital (D)	LC-MS/MS	17.1 (1.33)	17.8 (5.19)	10.3	28.4
Serum PLP, nmol/L	Bevital (D)	LC-MS/MS	54.5 (1.39)	57.4 (19.3)	26.6	109
Serum 4´-pyridoxic acid, nmol/L	Bevital (D)	LC-MS/MS	18.9 (1.26)	19.5 (4.86)	12.8	35.2
Serum riboflavin, nmol/L	Bevital (D)	LC-MS/MS	13.5 (1.76)	16.1 (12.0)	5.54	61.7
Serum thiamine, nmol/L	Bevital (D)	LC-MS/MS	7.34 (1.30)	7.59 (2.10)	5.00	13.4
Serum TMP, nmol/L	Bevital (D)	LC-MS/MS	3.04 (1.58)	3.36 (1.46)	1.05	7.07
**Routine clinical markers**						
Serum albumin, g/L	MBF	Photometry	45.3 (1.06)	45.4 (2.45)	40.0	49.0

	Laboratory (platform)[1]	Method	gMean (gSD)	Mean (SD)	Minimum	Maximum
Serum ALAT, U/L	MBF	Photometry	21.3 (1.43)	22.8 (10.7)	10.0	73.0
Serum ASAT, U/L	MBF	Photometry	24.0 (1.43)	25.9 (12.7)	16.0	74.0
Serum creatine, µmol/L	Bevital (C)	LC-MS/MS	19.2 (1.38)	20.2 (7.18)	10.2	42.2
Serum creatinine, µmol/L	MBF	Photometry	74.8 (1.19)	76.0 (13.8)	52.0	112
Serum creatinine, µmol/L	Bevital (C)	LC-MS/MS	78.9 (1.18)	80.1 (14.1)	58.8	120
Serum CRP, mg/L	MBF	Immuno-turbidimetry	0.86 (2.57)	1.41 (1.85)	0.16	9.00
Erythrocytes, 10^{12}/L	MBF	Flow cytometry	4.57 (1.09)	4.59 (0.40)	3.90	5.40
Serum gamma-glutamyltransferase, U/L	MBF	Photometry	16.0 (1.53)	17.9 (10.9)	9.00	65.0
Capillary glucose, mmol/L	-	Fingerprick, HemoCue 201 RT	5.27 (1.10)	5.29 (0.49)	4.50	6.40
Hemoglobin, g/dL	MBF	Photometry	13.8 (1.09)	13.8 (1.21)	11.8	17.3
HbA1c, mmol/mol	MBF	Immuno-agglutination	31.3 (1.11)	31.4 (3.10)	24.0	39.0
Serum insulin, mIU/L	MBF	Immunoassay	4.45 (1.94)	5.34 (3.0)	1.0	11.2
Mean corpuscular hemoglobin, pg	MBF	Calculated	30.1 (1.05)	30.2 (1.54)	26.0	34.0
Mean corpuscular volume, fL	MBF	Flow cytometry	89.8 (1.04)	89.9 (3.82)	81.0	100
Mean platelet volume, fL	MBF	Flow cytometry	10.4 (1.09)	10.5 (0.88)	9.00	12.7
Thrombocytes, 10^{9}/L	MBF	Impedance and flow cytometry	230 (1.22)	234 (45.4)	153	354
Serum TSH, mIU/L	MBF	Immunoassay	2.39 (1.58)	2.63 (1.09)	0.79	5.06
Serum 25-hydroxyvitamin D, nmol/L	MBF	Immunoassay	65.4 (1.33)	67.9 (18.9)	38.0	109
Serum HDL cholesterol, mmol/L	MBF	Photometry	1.55 (1.24)	1.58 (0.36)	1.00	2.80

	Laboratory (platform)[1]	Method	gMean (gSD)	Mean (SD)	Minimum	Maximum
Serum LDL cholesterol, mmol/L	MBF	Photometry	2.51 (1.31)	2.61 (0.72)	1.60	4.30
Serum triglycerides, mmol/L	MBF	Photometry	0.85 (1.46)	0.92 (0.36)	0.50	1.82
Serum magnesium, mmol/L	MBF	Photometry	0.81 (1.06)	0.82 (0.04)	0.73	0.91
Serum phosphate, mmol/L	MBF	Photometry	1.14 (1.13)	1.14 (0.13)	0.75	1.39
Serum potassium, mmol/L	MBF	Indirect ion-selective electrode	4.07 (1.07)	4.08 (0.28)	3.30	4.70

Abbreviations: gMean, geometric mean; gSD, geometric standard deviation; GC-MS/MS, Gas chromatography mass spectrometry; LC-MS/MS, Liquid chromatography mass spectrometry; DMG, Dimethylglycine; MBF, Department of Medical Biochemistry and Pharmacology, Haukeland University Hospital; FMN, Flavin mononucleotide; MMA, Methylmalonic acid; PLP, Pyridoxal-5´-phosphate; TMP, Thiamine monophosphate; ALAT, alanine aminotransferase; ASAT, aspartate aminotransferase; CRP, C-reactive protein; HbA1c, Glycated hemoglobin; TSH, Thyroid stimulating hormone; LDL, Low-density lipoprotein; HDL, High-density lipoprotein. [1]More information can be found at https://bevital.no/ (Bevital) and at https://helse-bergen.no/en/avdelinger/laboratorieklinikken/medisinsk-biokjemi-og-farmakologi (MBF).

Time-Resolved Metabolite Concentrations during the Postprandial and Fasting State: The Postprandial Metabolism in Healthy Young Adults (PoMet) study. *Åslaug Matre Anfinsen*

Supplementary Table 2. The metabolite concentrations after the consumption of a standardized meal in healthy subjects in the Postprandial Metabolism Study[1]

Serum biomarker	Time after meal													
	Ref	15 min	30 min	45 min	60 min	90 min	2 hours	3 hours	4 hours	6 hours	8 hours	10 hours	12 hours	24 hours
n	34	34	34	34	34	34	34	33	33	33	33	33	33	33
**Glucose and insulin**														
Glucose, mmol/L[2]	5.27	7.47	7.42	6.32	5.72	5.26	5.00	5.18	5.18	4.95	4.69	4.71	4.58	4.55
Insulin, mIU/L	4.45	37.7	41.3	32.5	26.8	17.6	12.1	7.38	3.81	2.42	2.07	1.89	1.98	2.00
**Amino acids**														
Alanine, µmol/L	332	417	452	469	472	468	447	397	358	309	295	287	292	334
Arginine, µmol/L	85.8	104	99.0	100	98.2	101	102	93.4	85.2	82.2	84.1	84.5	88.1	86.2
Asparagine, µmol/L	66.5	87.3	86.8	85.8	84.8	83.8	81.0	73.0	66.5	63.1	64.3	63.0	62.8	62.5
Aspartate, µmol/L	17.7	17.5	17.6	17.5	17.6	17.6	17.6	18.0	18.0	17.8	18.2	18.4	18.2	17.8
Glutamate, µmol/L	43.1	40.1	45.3	45.7	46.0	40.7	39.9	40.5	39.3	37.2	37.6	38.6	37.2	36.6
Glutamine, µmol/L	506	534	533	530	531	549	555	548	540	536	542	540	526	507
Histidine, µmol/L	80.1	86.8	88.2	87.9	87.8	90.5	92.5	88.6	83.0	79.0	79.4	79.6	80.6	84.6
Isoleucine, µmol/L	64.6	75.8	76.0	76.4	76.8	80.5	82.8	76.0	66.9	64.7	68.7	71.2	73.9	82.6
Leucine, µmol/L	128	147	147	148	148	154	159	148	135	133	141	146	149	176
Lysine, µmol/L	157	175	176	176	177	185	190	181	165	153	157	160	162	172
Phenylalanine, µmol/L	70.6	78.0	79.8	81.5	83.3	85.8	85.7	78.3	70.7	67.7	71.8	73.1	73.8	73.9
Proline, µmol/L	179	234	248	261	270	277	278	249	220	190	176	166	158	142
Sarcosine, µmol/L	1.35	1.56	1.59	1.60	1.63	1.68	1.69	1.55	1.38	1.21	1.14	1.09	1.04	0.93
Threonine, µmol/L	132	153	153	151	150	150	147	139	127	120	119	116	113	114
Tryptophan, µmol/L	71.3	74.3	72.7	72.2	72.2	72.7	73.0	68.4	62.0	58.3	62.7	65.3	68.6	63.5

Time-Resolved Metabolite Concentrations during the Postprandial and Fasting State: The Postprandial Metabolism in Healthy Young Adults (PoMet) study. *Åslaug Matre Anfinsen*

Serum biomarker	Time after meal													
	**Ref**	**15 min**	**30 min**	**45 min**	**60 min**	**90 min**	**2 hours**	**3 hours**	**4 hours**	**6 hours**	**8 hours**	**10 hours**	**12 hours**	**24 hours**
Tyrosine, µmol/L	62.4	67.9	69.5	70.9	72.9	77.3	80.6	76.4	67.0	56.2	54.8	53.7	53.8	56.3
Valine, µmol/L	250	267	270	270	270	277	284	278	265	255	253	254	256	285
**One-carbon metabolites**														
Betaine, µmol/L	33.0	36.6	38.2	39.1	39.8	41.3	41.3	37.9	36.4	34.3	34.2	32.5	31.8	30.3
Choline, µmol/L	9.19	10.4	10.2	9.96	9.89	9.59	9.63	9.53	9.35	9.18	9.67	8.96	8.52	8.71
Cystathionine, µmol/L	0.18	0.17	0.18	0.19	0.20	0.21	0.23	0.24	0.22	0.16	0.13	0.13	0.12	0.13
Cysteine, µmol/L	251	249	244	242	242	240	239	239	243	253	265	271	276	281
DMG, µmol/L	3.45	3.50	3.53	3.52	3.63	3.86	3.85	3.75	3.57	3.28	3.26	3.56	3.60	3.68
Glycine, µmol/L	257	273	271	268	267	273	271	258	251	244	247	247	245	248
tHcy, µmol/L	8.16	8.00	7.89	7.83	7.89	7.88	7.91	8.07	8.16	8.27	8.56	8.75	8.83	9.06
Methionine, µmol/L	29.3	33.9	34.2	34.8	35.4	37.2	37.9	33.4	28.0	25.2	26.6	26.9	27.2	27.9
MetSo, µmol/L	0.78	0.89	0.94	1.11	1.20	1.31	1.33	1.29	0.97	0.77	0.69	0.64	0.70	0.66
Serine, µmol/L	131	150	151	150	149	148	147	139	132	129	132	133	133	130
**B-vitamin markers**														
Cobalamin, pmol/L	328	322	320	319	323	321	316	330	335	336	342	351	353	379
FMN, nmol/L	8.07	6.51	6.06	5.72	5.64	5.93	6.27	7.22	8.46	10.5	11.4	11.8	12.4	15.1
Folate, nmol/L	13.8	15.4	15.7	15.7	15.6	14.9	14.4	14.3	14.6	16.3	17.5	18.0	18.2	22.0
nNAM	106	128	125	117	115	117	115	106	106	113	114	97	77	108
MMA, µmol/L	0.14	0.14	0.14	0.14	0.14	0.14	0.15	0.14	0.13	0.12	0.12	0.11	0.12	0.12
Nicotinamide, nmol/L	203	281	252	219	191	176	164	160	185	194	188	172	138	184
Pyridoxal, nmol/L	17.1	18.9	19.3	19.4	19.3	18.4	18.2	16.9	16.4	16.2	16.0	16.1	16.1	15.6
PLP, nmol/L	54.5	52.8	51.6	50.3	49.6	48.8	48.3	48.1	48.0	47.1	46.2	44.6	43.4	45.9
Pyridoxic acid, nmol/L	18.9	18.4	17.3	15.5	15.3	14.3	13.9	14.2	14.7	14.8	14.2	14.3	13.9	19.6

Time-Resolved Metabolite Concentrations during the Postprandial and Fasting State: The Postprandial Metabolism in Healthy Young Adults (PoMet) study. *Åslaug Matre Anfinsen*

Serum biomarker	Time after meal													
	**Ref**	**15 min**	**30 min**	**45 min**	**60 min**	**90 min**	**2 hours**	**3 hours**	**4 hours**	**6 hours**	**8 hours**	**10 hours**	**12 hours**	**24 hours**
Riboflavin, nmol/L	13.5	13.4	13.6	13.8	13.9	13.4	13.3	12.6	12.8	13.6	13.4	14.7	14.9	17.6
Thiamine, nmol/L	7.34	8.77	9.09	8.88	8.75	8.43	8.00	7.68	7.55	7.21	7.14	7.06	6.54	5.84
TMP, nmol/L	3.05	3.39	3.19	3.19	3.02	2.93	2.92	3.31	3.51	3.49	3.17	2.97	2.87	2.68

Abbreviations: DMG, Dimethylglycine; FMN, Flavin mononucleotide; MetSo, Methionine sulfoxide; mNAM, N^1-methylnicotinamide; MMA, Methylmalonic acid; PLP, Pyridoxal-5´-phosphate; tHcy, Total homocysteine; TMP, Thiamine monophosphate. [1]All values are reported as geometric means [2]Capillary glucose.

Supplementary Table 3. The metabolite concentrations after the consumption of a standardized meal in male (*n*= 18) and female (*n* = 16) participants in the Postprandial Metabolism Study[1]

		Time after meal													
**Serum biomarker**	**Sex**[2]	**Ref**	**15 min**	**30 min**	**45 min**	**60 min**	**90 min**	**2 hours**	**3 hours**	**4 hours**	**6 hours**	**8 hours**	**10 hours**	**12 hours**	**24 hours**
n		34	34	34	34	34	34	34	33	33	33	33	33	33	33
**Glucose and insulin**															
Glucose, mmol/L[3]	**M**	5.40	7.59	7.65	6.56	5.78	5.16	5.05	5.11	5.08	4.95	4.68	4.66	4.68	4.59
	**F**	5.12	7.33	7.17	6.06	5.64	5.37	4.95	5.27	5.29	4.94	4.69	4.77	4.46	4.51
Insulin, mIU/L	**M**	4.53	36.5	41.2	32.7	26.4	17.3	12.3	7.27	3.36	1.97	1.93	1.59	1.69	1.76
	**F**	4.37	39.0	41.3	32.4	27.2	18.0	11.9	7.51	4.43	3.11	2.24	2.33	2.39	2.33
**Amino acids**															
Alanine, µmol/L	**M**	342	425	459	474	479	472	457	405	368	320	308	304	303	343
	**F**	321	409	444	463	465	462	436	388	346	296	279	268	279	325
Arginine, µmol/L	**M**	87.5	107	102	103	102	104	104	94.2	86.2	83.8	85.8	86.2	89.0	87.1
	**F**	84.0	101	95.4	97.5	94.4	98.4	100	92.5	83.9	80.3	82.2	82.5	87.1	85.2
Asparagine, µmol/L	**M**	68.7	89.0	89.5	87.9	86.7	85.0	82.7	73.4	67.1	64.6	65.7	64.7	64.0	66.1
	**F**	64.0	85.4	83.9	83.5	82.7	82.6	79.1	72.4	65.7	61.2	62.7	61.0	61.3	58.5
Aspartate, µmol/L	**M**	16.1	16.0	16.3	16.1	16.3	16.3	16.1	16.4	16.3	16.1	16.6	16.8	16.7	16.5
	**F**	19.6	19.3	19.2	19.1	19.2	19.2	19.3	20.2	20.3	20.2	20.3	20.6	20.2	19.5
Glutamate, µmol/L	**M**	46.4	44.0	49.5	48.3	49.3	43.0	41.8	42.8	40.5	39.5	38.7	40.0	40.9	38.5
	**F**	39.6	36.1	40.9	43.0	42.6	38.2	37.8	38.0	37.8	34.6	36.4	37.0	33.3	34.3
Glutamine, µmol/L	**M**	551	582	585	583	582	598	608	593	582	584	592	584	565	560
	**F**	459	485	480	477	479	498	500	498	495	484	488	492	483	451
Histidine, µmol/L	**M**	79.4	85.5	87.8	87.9	87.4	89.6	91.9	86.9	81.3	78.5	78.9	78.9	79.8	85.2

		Time after meal													
**Serum biomarker**	**Sex[2]**	**Ref**	**15 min**	**30 min**	**45 min**	**60 min**	**90 min**	**2 hours**	**3 hours**	**4 hours**	**6 hours**	**8 hours**	**10 hours**	**12 hours**	**24 hours**
n		34	34	34	34	34	34	34	33	33	33	33	33	33	33
	**F**	80.8	88.2	88.7	88.0	88.2	91.7	93.1	90.6	85.1	79.7	80.0	80.4	81.7	83.8
Isoleucine, µmol/L	**M**	68.5	80.9	81.6	81.6	81.9	83.5	84.4	74.9	65.6	65.7	70.6	72.8	75.5	83.0
	**F**	60.4	70.5	70.1	70.8	71.5	77.2	81.1	77.3	68.6	63.5	66.4	69.2	72.1	82.1
Leucine, µmol/L	**M**	136	156	157	158	158	161	163	148	134	137	146	151	154	181
	**F**	120	137	136	137	138	147	154	148	135	128	134	141	144	169
Lysine, µmol/L	**M**	162	182	183	184	186	192	196	184	168	159	163	165	168	180
	**F**	151	168	167	166	167	178	182	177	161	147	150	153	156	162
Phenylalanine, µmol/L	**M**	70.3	77.8	79.5	80.9	82.3	83.7	82.8	73.9	67.0	66.5	71.3	73.0	73.7	73.7
	**F**	71.0	78.2	80.1	82.1	84.6	88.3	89.1	84.0	75.4	69.3	72.3	73.2	73.9	74.2
Proline, µmol/L	**M**	203	258	272	282	290	293	297	267	240	215	203	193	184	166
	**F**	155	209	223	238	250	260	259	229	198	164	149	139	131	118
Sarcosine, µmol/L	**M**	1.52	1.77	1.81	1.82	1.84	1.87	1.88	1.72	1.55	1.39	1.31	1.25	1.20	1.08
	**F**	1.18	1.35	1.37	1.39	1.42	1.49	1.50	1.37	1.20	1.03	0.97	0.92	0.88	0.78
Threonine, µmol/L	**M**	140	159	161	160	157	155	154	143	131	126	124	122	117	121
	**F**	125	146	145	141	142	144	140	133	122	113	112	110	108	106
Tryptophan, µmol/L	**M**	75.1	78.4	76.6	76.0	76.0	75.8	75.5	69.9	64.1	61.2	65.0	69.3	72.1	66.1
	**F**	67.3	70.0	68.6	68.2	68.3	69.4	70.2	66.6	59.5	55.0	60.0	60.8	64.6	60.4
Tyrosine, µmol/L	**M**	65.6	71.8	73.7	75.1	77.0	80.2	82.7	76.8	67.8	59.3	58.2	57.1	57.2	58.6
	**F**	58.9	63.7	64.9	66.4	68.6	74.2	78.2	75.9	66.1	52.8	51.0	49.9	50.1	53.7
Valine, µmol/L	**M**	259	277	281	282	282	286	292	281	265	259	259	260	262	291
	**F**	241	257	259	257	257	267	276	276	264	250	246	247	249	279

Time-Resolved Metabolite Concentrations during the Postprandial and Fasting State: The Postprandial Metabolism in Healthy Young Adults (PoMet) study. *Åslaug Matre Anfinsen*

**Serum biomarker**	**Sex[2]**	**Time after meal**													
		**Ref**	**15 min**	**30 min**	**45 min**	**60 min**	**90 min**	**2 hours**	**3 hours**	**4 hours**	**6 hours**	**8 hours**	**10 hours**	**12 hours**	**24 hours**
n		34	34	34	34	34	34	34	33	33	33	33	33	33	33
**One-carbon metabolites**															
Betaine, µmol/L	**M**	37.6	41.7	43.9	45.1	45.3	46.3	46.3	43.1	41.6	38.4	38.4	36.6	36.2	36.4
	**F**	28.6	31.6	32.6	33.3	34.3	36.4	36.2	32.6	31.0	29.9	29.7	28.1	27.1	24.2
Choline, µmol/L	**M**	9.34	10.5	10.3	10.1	10.1	9.64	9.70	9.61	9.65	9.42	9.97	9.25	9.17	9.07
	**F**	9.04	10.4	10.0	9.85	9.68	9.53	9.55	9.44	9.01	8.90	9.34	8.61	7.81	8.30
Cystathionine, µmol/L	**M**	0.18	0.18	0.19	0.20	0.21	0.23	0.25	0.26	0.23	0.17	0.15	0.14	0.14	0.14
	**F**	0.17	0.17	0.18	0.18	0.18	0.20	0.22	0.23	0.21	0.15	0.12	0.11	0.11	0.12
Cysteine, µmol/L	**M**	257	254	250	246	246	245	245	244	248	258	270	276	282	287
	**F**	244	244	238	237	237	235	232	233	237	246	259	265	269	273
DMG, µmol/L	**M**	3.66	3.89	3.80	3.75	3.96	4.23	4.17	4.15	3.92	3.58	3.48	3.83	3.86	3.91
	**F**	3.22	3.11	3.24	3.28	3.30	3.48	3.51	3.32	3.20	2.95	3.00	3.26	3.31	3.43
Glycine, µmol/L	**M**	260	279	280	278	277	281	281	266	258	252	253	253	249	256
	**F**	253	267	262	258	257	263	260	248	243	235	240	240	240	239
tHcy, µmol/L	**M**	8.69	8.55	8.42	8.31	8.38	8.45	8.53	8.68	8.75	8.90	9.27	9.44	9.58	9.82
	**F**	7.60	7.42	7.33	7.32	7.37	7.28	7.27	7.40	7.50	7.57	7.77	8.00	8.01	8.23
Methionine, µmol/L	**M**	37.6	41.7	43.9	45.1	45.3	46.3	46.3	43.1	41.6	38.4	38.4	36.6	36.2	36.4
	**F**	27.8	32.0	32.1	32.7	33.4	35.5	36.5	33.5	27.9	24.0	25.2	25.4	25.7	26.5
MetSo, µmol/L	**M**	0.74	0.79	0.86	0.99	1.06	1.12	1.23	1.10	0.82	0.73	0.61	0.63	0.69	0.59
	**F**	0.82	1.02	1.03	1.25	1.37	1.55	1.46	1.55	1.18	0.82	0.80	0.65	0.71	0.74
Serine, µmol/L	**M**	129	147	150	149	147	145	144	134	128	126	128	129	129	130
	**F**	133	152	153	151	151	151	150	145	138	133	138	138	137	131

Time-Resolved Metabolite Concentrations during the Postprandial and Fasting State: The Postprandial Metabolism in Healthy Young Adults (PoMet) study. *Åslaug Matre Anfinsen*

Serum biomarker	Sex[2]	Time after meal Ref	15 min	30 min	45 min	60 min	90 min	2 hours	3 hours	4 hours	6 hours	8 hours	10 hours	12 hours	24 hours
n		34	34	34	34	34	34	34	33	33	33	33	33	33	33
**B-vitamin markers**															
Cobalamin, pmol/L	**M**	330	322	319	314	323	319	312	325	333	332	336	346	348	373
	**F**	325	321	322	326	323	322	321	336	336	341	350	358	360	387
FMN, nmol/L	**M**	7.75	5.96	5.57	5.56	5.30	5.47	5.82	6.66	7.86	9.93	11.4	11.2	12.3	14.9
	**F**	8.44	7.19	6.65	5.91	6.06	6.50	6.82	7.94	9.24	11.3	11.4	12.7	12.5	15.4
Folate, nmol/L	**M**	14.0	15.8	16.0	16.1	15.9	15.1	14.5	14.6	14.8	16.8	18.0	18.1	17.9	21.1
	**F**	13.6	15.0	15.3	15.2	15.2	14.7	14.3	14.0	14.5	15.8	16.8	18.0	18.6	23.1
mNAM, nmol/L	**M**	90.6	107	105	100	97.5	103	106	97	102	110	109	93.8	70.8	96.6
	**F**	126	157	151	139	139	135	125	119	112	118	120	102	85	124
MMA, µmol/L	**M**	0.13	0.13	0.13	0.14	0.14	0.14	0.14	0.14	0.13	0.12	0.11	0.11	0.11	0.12
	**F**	0.14	0.15	0.14	0.15	0.14	0.15	0.15	0.15	0.14	0.12	0.12	0.11	0.12	0.13
Nicotinamide, nmol/L	**M**	194	296	282	257	220	205	189	184	208	221	205	185	136	171
	**F**	214	265	222	182	163	148	140	135	162	166	170	157	141	200
Pyridoxal, nmol/L	**M**	18.9	20.6	20.9	21.0	20.6	19.7	19.5	18.0	18.0	17.7	17.4	17.6	17.5	17.3
	**F**	15.2	17.1	17.6	17.8	17.9	17.0	16.9	15.8	14.5	14.6	14.6	14.5	14.5	13.8
PLP, nmol/L	**M**	61.9	60.0	58.9	57.3	56.7	54.9	55.0	54.1	54.5	53.8	53.0	51.7	50.9	53.9
	**F**	47.2	45.7	44.5	43.5	42.8	42.7	41.7	41.7	41.3	40.3	39.2	37.3	35.8	37.9
Pyridoxic acid, nmol/L	**M**	19.6	18.8	18.1	16.2	16.1	15.1	14.8	15.1	16.2	16.5	15.1	15.4	14.7	20.2
	**F**	18.2	17.8	16.5	14.7	14.4	13.4	12.9	13.2	13.1	13.1	13.2	13.1	13.0	18.8
Riboflavin, nmol/L	**M**	11.2	11.1	11.4	11.6	11.5	11.4	10.8	10.6	10.5	11.4	11.2	12.2	12.7	14.5
	**F**	16.5	16.5	16.6	16.8	17.2	16.1	16.8	15.5	16.1	16.7	16.7	18.3	18.0	22.2

Time-Resolved Metabolite Concentrations during the Postprandial and Fasting State: The Postprandial Metabolism in Healthy Young Adults (PoMet) study. *Åslaug Matre Anfinsen*

**Serum biomarker**	**Sex[2]**	**Ref**	**15 min**	**30 min**	**45 min**	**60 min**	**90 min**	**2 hours**	**3 hours**	**4 hours**	**6 hours**	**8 hours**	**10 hours**	**12 hours**	**24 hours**
		**Time after meal**													
n		34	34	34	34	34	34	34	33	33	33	33	33	33	33
Thiamine, nmol/L	**M**	7.01	8.52	8.73	8.53	8.42	8.09	7.70	7.17	7.12	6.95	6.82	6.91	6.50	5.76
	**F**	7.73	9.06	9.50	9.29	9.14	8.82	8.34	8.36	8.10	7.52	7.54	7.25	6.59	5.94
TMP, nmol/L	**M**	3.04	3.44	3.28	3.23	3.08	3.11	3.11	3.39	3.63	3.58	3.43	3.06	3.22	2.81
	**F**	3.05	3.34	3.10	3.15	2.95	2.73	2.71	3.21	3.37	3.38	2.88	2.86	2.50	2.53

Abbreviations: DMG, Dimethylglycine; FMN, Flavin mononucleotide; MetSo, Methionine sulfoxide; mNAM, N^1-methylnicotinamide; MMA, Methylmalonic acid; PLP, Pyridoxal-5´-phosphate; tHcy, Total homocysteine; TMP, Thiamine monophosphate. [1]All values are reported as geometric means [2]Male/Female [3]Capillary glucose.

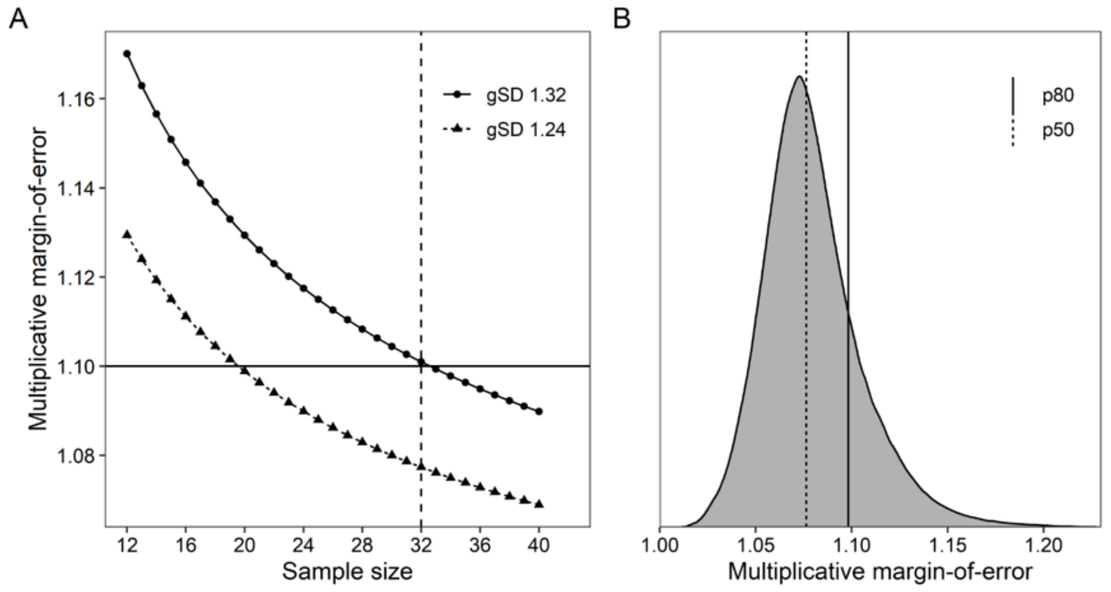

Supplementary Figure 1. A) Precision curves as a function of sample size, using the observed median (20th-80th percentile) geometric standard deviations from the HuMet study (available from http://metabolomics.helmholtz-muenchen.de/humet/). **B)** The expected distribution of multiplicative margin-of-errors for the measurements of different metabolites at different time points, with *n* = 32. We expected to be able to estimate the geometric mean concentrations within a multiplicative margin-of-error of 1.10 for at least 80% of all measurements. **Abbreviations:** gSD, geometric standard deviation

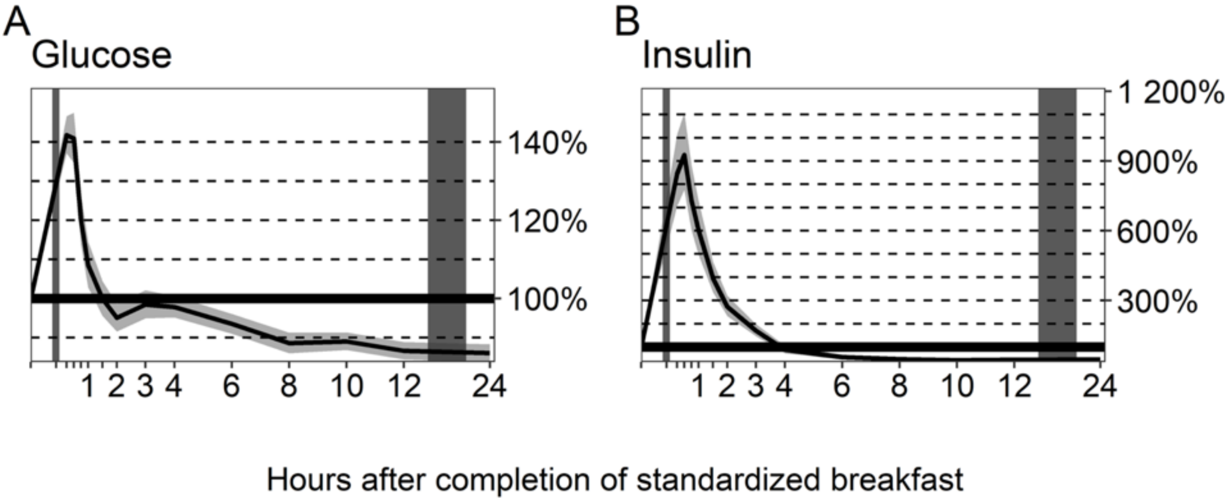

Supplementary Figure 2. The relative change in glucose- and insulin concentrations (% change from reference values) as a function of time since completion of the standardized breakfast meal in participants in the Postprandial Metabolism Study (*n* = 34). The solid black line represents the geometric mean, while the grey shaded area represents the 95% geometric confidence intervals. The leftmost vertical line indicates the time of the standardized breakfast meal, while the rightmost vertical line indicates time spent outside the study center.

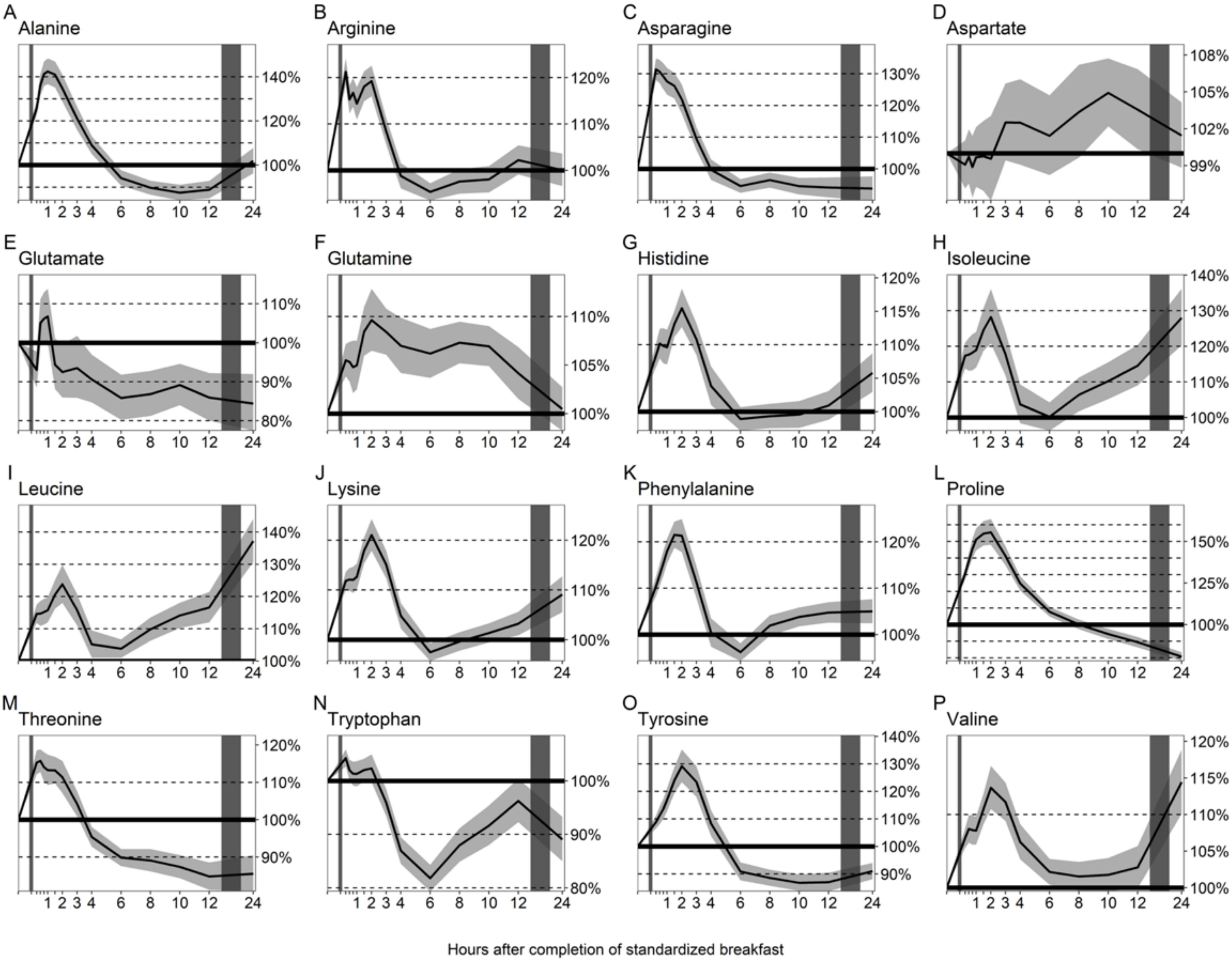

Supplementary Figure 3. The relative change in amino acid concentrations (% change from reference values) as a function of time since completion of the standardized breakfast meal in participants in the Postprandial Metabolism Study (*n* = 34). The solid black line represents the geometric mean, while the grey shaded area represents the 95% geometric confidence intervals. The leftmost vertical line indicates the time of the standardized breakfast meal, while the rightmost vertical line indicates time spent outside the study center.

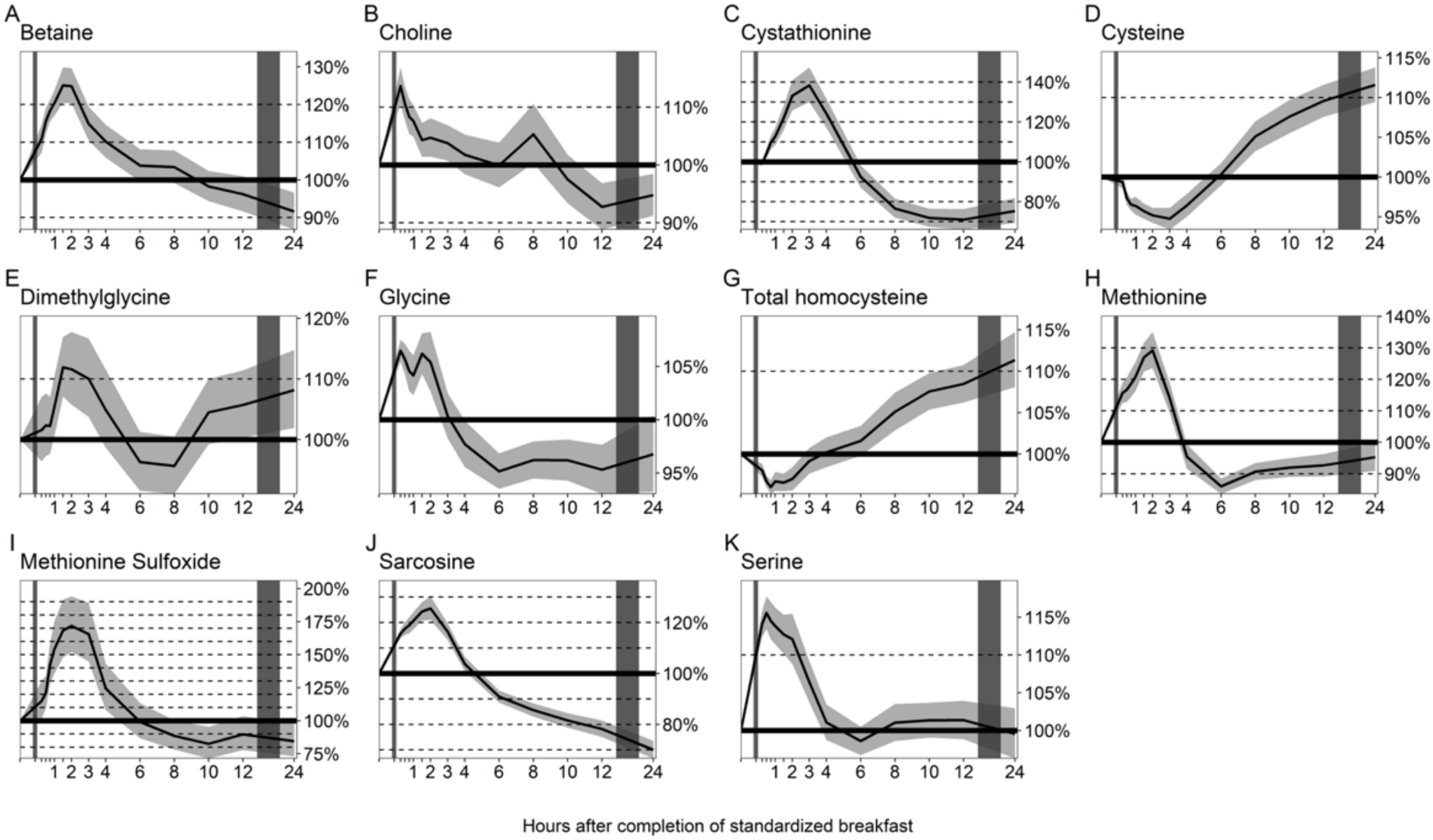

Supplementary Figure 4. The relative change in one-carbon metabolite concentrations (% change from reference values) as a function of time since completion of the standardized breakfast meal in participants in the Postprandial Metabolism Study (*n* = 34). The solid black line represents the geometric mean, while the grey shaded area represents the 95% geometric confidence intervals. The leftmost vertical line indicates the time of the standardized breakfast meal, while the rightmost vertical line indicates time spent outside the study center.

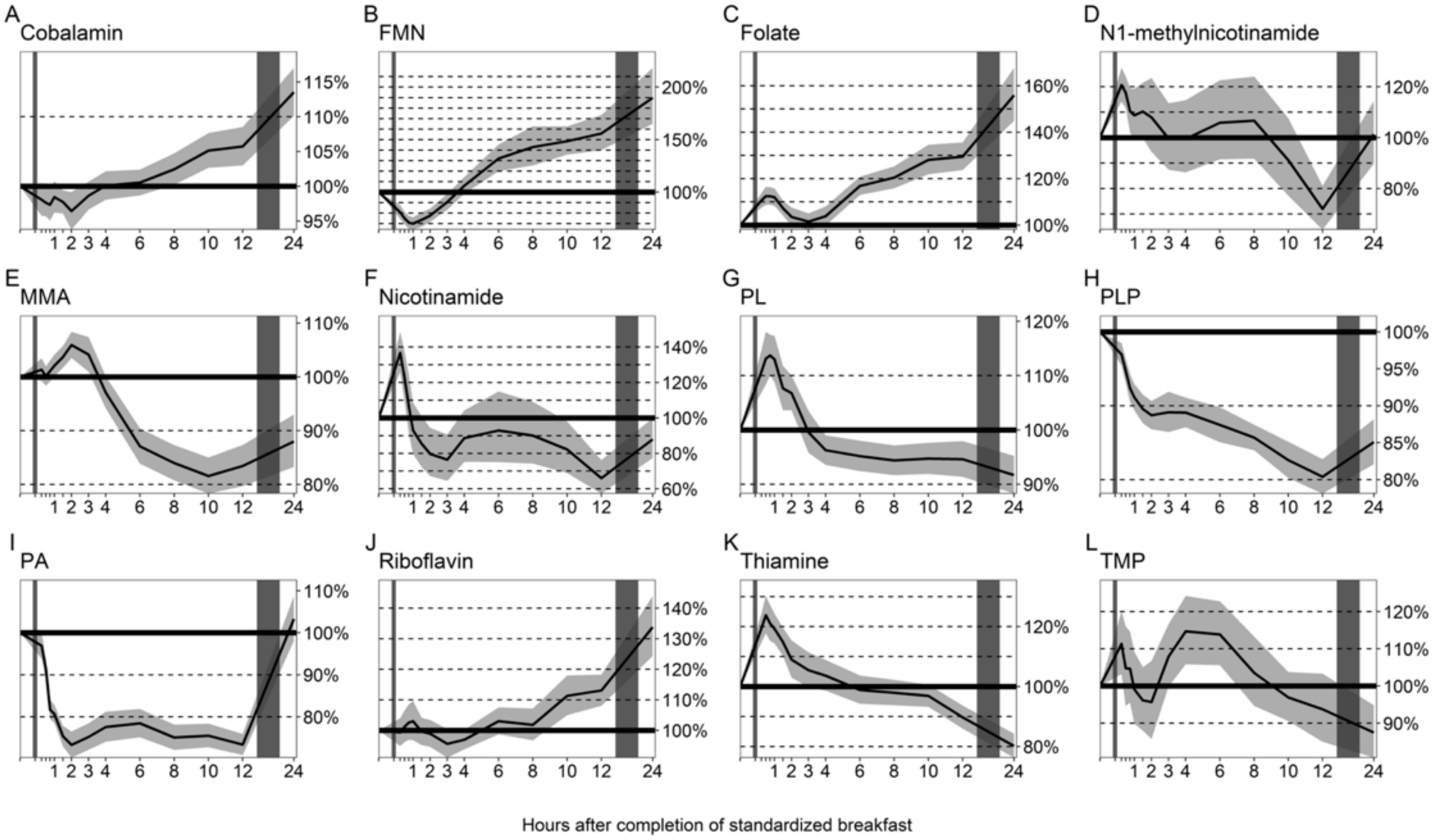

Supplementary Figure 5. The relative change in B-vitamin biomarker concentrations (% change from reference values) as a function of time since completion of the standardized breakfast meal in participants in the Postprandial Metabolism Study (*n* = 34). The solid black line represents the geometric mean, while the grey shaded area represents the 95% geometric confidence intervals. The leftmost vertical line indicates the time of the standardized breakfast meal, while the rightmost vertical line indicates time spent outside the study center.

Abbreviations: FMN, Flavin mononucleotide; MMA, methylmalonic acid; PA, 4´-pyridoxic acid; PL, Pyridoxal; PLP, Pyridoxal-5´-phosphate
